# The CSF1^+^ tumor cell–SPP1^+^ macrophage axis drives gastric cancer progression and immunotherapy resistance

**DOI:** 10.3389/fimmu.2026.1817573

**Published:** 2026-06-16

**Authors:** Heng Zhang, Yundong You, Jinhao Peng, Qinhao Du, Xifan Liu

**Affiliations:** Department of Gastrointestinal Surgery, Xiangyang Central Hospital, Affiliated Hospital of Hubei University of Arts and Science, Xiangyang, Hubei, China

**Keywords:** CSF1–CSF1R, gastric cancer, immunotherapy resistance, SPP1+ macrophages, tumor microenvironment

## Abstract

**Background:**

Gastric cancer (GC) remains a leading cause of global cancer mortality, characterized by profound heterogeneity. While immune checkpoint blockade (ICB) has emerged as a promising strategy, pervasive resistance frequently limits its clinical efficacy. Elucidating the mechanisms driving this resistance and identifying predictive biomarkers remain critical challenges for achieving precision oncology in GC.

**Methods:**

We constructed a spatial multi-omic atlas by integrating scRNA-seq from GC patients with public spatial transcriptomics (ST) data. Computational deconvolution of independent immunotherapy cohorts was performed to pinpoint specific macrophage and fibroblast subsets linked to ICB efficacy. Intercellular immunosuppressive signaling and spatial proximity were characterized via cell-cell communication and ST analysis. We validated the biological significance of this crosstalk using non-contact co-culture systems and mIHC analysis of an independent clinical cohort.

**Results:**

We constructed a spatially resolved multi-omic atlas by integrating scRNA-seq from 131,027 cells with spatial transcriptomics. Deconvolution of immunotherapy cohorts identified the synchronous enrichment of SPP1+macrophages and MFAP5+fibroblasts in tumors with poor ICB efficacy. Spatial analysis quantified a significant co-localization between CSF1-producing malignant cells and SPP1^+^ macrophages. Experimentally, malignant cell-derived CSF1 induced SPP1+ macrophage polarization, triggering the synergistic upregulation of *IL-10* and *TGF-β1*. This crosstalk significantly enhanced GC cell colony formation and invasive potential, as confirmed by secretome profiling and functional assays. Finally, mIHC staining validated the co-localization of CSF1+malignant cells and SPP1+macrophages *in situ* within clinical tumor tissues.

**Conclusions:**

Our study identifies a spatially organized niche involving CSF1+malignant cells and SPP1+ macrophages as a key driver of ICB resistance in GC. By defining the CSF1-mediated crosstalk between these two cell types, these findings highlight promising therapeutic targets to enhance the efficacy of immunotherapy.

## 1 Introduction

Gastric cancer (GC) is the fifth most common malignancy and the third leading cause of cancer-related death worldwide (1–3). Despite advances in surgery, chemotherapy, radiotherapy, targeted therapy, and immunotherapy, the prognosis remains poor, with a 5-year survival rate of less than 40% (4–7). While immune checkpoint blockade has revolutionized oncology, its application in GC is marked by limited success which includes low objective response rates and the frequent emergence of resistance. These challenges underscore the urgent need to decipher the underlying mechanisms of immune evasion within the GC microenvironment to develop more effective therapeutic strategies (8).

The tumor microenvironment (TME), particularly stromal cells such as cancer-associated fibroblasts (CAFs) and myeloid-derived immune cells such as tumor-associated macrophages (TAMs), plays a pivotal role in driving resistance to immunotherapy by fostering an immunosuppressive niche. Critically, the functional states and interactions of these cellular components are not uniform but are governed by precise spatial organization within the tumor tissue. Therefore, deciphering spatial heterogeneity-the specific anatomical positioning of and physical relationships between distinct cell types is fundamental to understanding the mechanisms underlying therapeutic resistance (9).

The advent of scRNA-seq and spatial transcriptomics has revolutionized the study of GC, providing unprecedented resolution to dissect tumor heterogeneity and deconstruct the multicellular ecosystem of the TME (10–13). These technologies have enabled the systematic identification of functionally distinct cellular subpopulations and the mapping of their spatial architecture within tissues, offering crucial insights into the cellular basis of disease progression and therapeutic resistance (14). For example, scRNA-seq analyses have revealed a complex immune landscape in GC, encompassing immunosuppressive TAMs, functionally exhausted CD8+ T cells, and myeloid-derived suppressor cells (MDSCs) (15). TAMs are frequently characterized by an immunosuppressive phenotype characterized by PD-L1, MARCO, and CD163 expression, which aids immune evasion, whereas exhausted T cells exhibit markers such as HAVCR2 and LAG3, which are associated with poor cytotoxic function and clinical outcomes. Spatial transcriptomics further contextualizes these findings, revealing organized cellular niches where cancer-associated fibroblasts (CAFs) expressing chemokines colocalize with and activate protumorigenic JAK-STAT3 signaling in macrophages, thereby reinforcing an immunosuppressive milieu. The integration of scRNA-seq with spatial data underscores that GC pathogenesis is orchestrated through spatially coordinated interactions between specific stromal and immune subsets. Understanding these cellular crosstalk networks is essential for identifying new, mechanism-based targets for cancer therapy.

In this study, we performed an integrated multi-omic analysis to systematically map the cellular architecture and communication networks within the GC TME. By combining scRNA-seq and spatial transcriptomics, we identified the spatial determinants of immune evasion and uncovered novel therapeutic targets to overcome immunotherapy resistance.

## 2 Materials and methods

### 2.1 Data acquisition and preprocessing

All single-cell RNA sequencing (scRNA-seq) and spatial transcriptomics (ST-seq) datasets utilized in this study were retrieved from publicly available repositories. Specifically, scRNA-seq data were obtained from human gastric cancer (GC) cohorts (GSE167297 and GSE163558), and ST-seq data were acquired from the GEO database (GSE251950). Furthermore, bulk RNA-seq datasets, including PRJEB25780 and the TCGA-STAD cohort, were integrated for downstream analysis. Prior to comprehensive analysis, all raw sequencing matrices underwent standard quality control, including the removal of low-quality cells and normalization, consistent with the protocols established in the original studies.

### 2.2 Analysis of scRNA-seq data

The R package Seurat (v4.3.0) was used for single-cell data analysis (16, 17). The merged scRNA-seq dataset was first normalized via a log-transformation approach, followed by the identification of 2,000 highly variable genes through the FindVariableFeatures function. Gene expression values were subsequently scaled via ScaleData, and batch effects across samples were corrected via the Harmony algorithm to achieve integrated clustering (18). Dimensionality reduction was then performed via principal component analysis (PCA) on the selected variable genes. The top 20 principal components were retained and used for downstream clustering through FindNeighbors and FindClusters (resolution = 0.5). To visualize the global transcriptomic landscape, uniform manifold approximation and projection (UMAP) was applied on the basis of the top 20 PCs. Cell type–specific markers were identified for 9 subclusters via the FindAllMarkers function, with thresholds set at log fold change > 0.5, minimum detection rate (min.pct) > 0.25, and adjusted p < 0.05 to ensure robust marker selection.

### 2.3 Analysis of the bulk RNA-seq data

The raw bulk RNA-seq data were filtered to remove genes expressed at low levels, normalized via DESeq2 (1.48.2), and analyzed for differential expression, with batch effects included when applicable (19). Differentially expressed genes (DEGs) were defined as those with |log2FC|≥ 1 and FDR < 0.05, with effect sizes refined by apparent shrinkage. Gene set enrichment analysis (GSEA) was performed with fgsea/clusterProfiler via MSigDB hallmark, KEGG and GO gene sets, and genes were ranked by DESeq2 statistics with 1,000 permutations (20, 21). Enriched pathways were considered significant at adjusted p < 0.05, and visualizations were generated with ggplot2.

### 2.4 Estimation of pathway and signature activity

Pathway-level activity was inferred via the gene set variation analysis (GSVA) algorithm, which calculates enrichment scores to represent the relative activation state of predefined biological processes (20). Cancer hallmark signatures were evaluated on the basis of curated gene sets obtained from the Molecular Signatures Database (MSigDB) (21, 22).

### 2.5 Fibroblast sub-clustering and annotation

To characterize fibroblast heterogeneity, 11,753 fibroblasts were extracted and re-analyzed using Seurat. Following batch effect correction via harmony dimensionality reduction was performed using the top 20 PCs. Based on an optimized resolution of 0.4, five stable transcriptional states were identified. Cluster-specific markers were determined using the FindAllMarkers function (Wilcoxon rank-sum test, logFC > 0.25, padj. p < 0.05). These sub-clusters were then annotated as POSTN+, MFAP5+, B2M+, and BGN+ and FABP3+ states by integrating DEGs.

### 2.6 Pseudotime analysis via monocle3

Single-cell RNA-seq data were preprocessed and normalized via Seurat, and the top 2,000 highly variable genes were selected for trajectory analysis. A Monocle3 CellDataSet object was created, followed by dimensionality reduction with PCA and UMAP (preprocess_cds and reduce_dimension) (23). The cells were clustered (cluster_cells), and the principal graph was learned (learn_graph) to infer lineage relationships. Pseudotime was assigned using early-stage cells as the root (order_cells), and gene expression dynamics along the trajectory were analyzed (graph_test). Trajectories and dynamic gene expression were visualized with plot_genes_in_pseudotime.

### 2.7 Cell–cell communication analysis

Cell–cell communication networks were inferred via the CellCall R package (v1.0.0) (24). The normalized expression matrices and cell-type annotations from Seurat were used to generate a CellCall object (Org = “Homo sapiens”, data_source = “UMI”). Ligand–receptor–transcription factor interactions were inferred via TransCommuProfile with the parameters pvalueCor = 0.05, Corvalue = 0.1, and method = “weighted”. Significant interactions (adjusted p < 0.05) were visualized via bubble plots and Circos diagrams to map the intercellular signaling network.

### 2.8 Immune cell composition estimation

The bulk transcriptomic data were deconvoluted via CIBERSORTx to estimate the proportions of immune cell subsets in the TME. Group differences in immune infiltration were assessed, and correlations between the risk score and immune cell fractions were calculated via Spearman’s rank correlation. Visualizations were created with ggplot2.

### 2.9 Survival analysis

Patient survival was analyzed via the survival package in R. Cox proportional hazards models were used to calculate hazard ratios (HRs) and 95% confidence intervals. Kaplan–Meier curves were generated with survival curves, and significance was determined via two-sided log-rank tests. Optimal thresholds for continuous variables, such as gene expression or immune infiltration, were determined via maxstat to maximize the rank statistic, enabling stratification of patients into high- and low-risk groups for downstream survival comparisons.

### 2.10 Statistical analysis

All data analyses were performed in R (version 4.2.0). Associations between variables were assessed via Spearman’s rank correlation, whereas group differences were evaluated via the Wilcoxon rank-sum test. A p value less than 0.05 was considered statistically significant unless stated otherwise.

### 2.11 Cell co-culture

To simulate the tumor microenvironment, a non-contact co-culture system was established using Transwell inserts with a 0.4 μm pore size. CSF1-expressing AGS cells were seeded in the upper chamber, while THP-1-derived macrophages were placed in the lower chamber. After 48h of co-culture, the supernatants were collected and centrifuged to remove cellular debris, generating co-culture conditioned medium (Co-CM). As a control, conditioned medium from macrophages cultured alone under identical conditions was collected and designated as NC-CM.

### 2.12 Cytokine quantification

The concentrations of key secreted factors, including *CSF1*, *IL-10*, *SPP1*, *TGFB1*, and *VEGFA*, were quantified in the conditioned media using commercial ELISA kits (Boster, China) according to the manufacturer’s instructions. Absorbance was measured at 450 nm using a microplate reader.

### 2.13 Functional characterization of AGS cells

Colony Formation Assay: AGS cells were seeded into 6-well plates at a density of 500 cells per well and cultured in either NC-CM or Co-CM for 14 days to evaluate long-term proliferative capacity. Colonies were fixed, stained, and counted.

Transwell Invasion Assay: For invasion assays, 3 × 10^4^ AGS cells were seeded into the upper chambers of Matrigel-coated Transwell inserts with 8 μm pore size in serum-free conditioned media. Medium supplemented with 10% FBS was added to the lower chamber as a chemoattractant. After 48 h, invaded cells were fixed, stained, and quantified.

### 2.14 Multiplex immunohistochemistry

Multiplex immunohistochemistry was performed on clinical gastric cancer tissue. The antibody panel included CD68 (macrophages), SPP1, EpCAM (tumor cells), and CSF1. Multispectral imaging was used to assess the spatial co-localization of CSF1^+^ malignant cells and SPP1^+^ macrophages.

## 3 Results

### 3.1 Single-cell and spatial transcriptomic profiling of the GC tumor microenvironment

**Figure 1:**
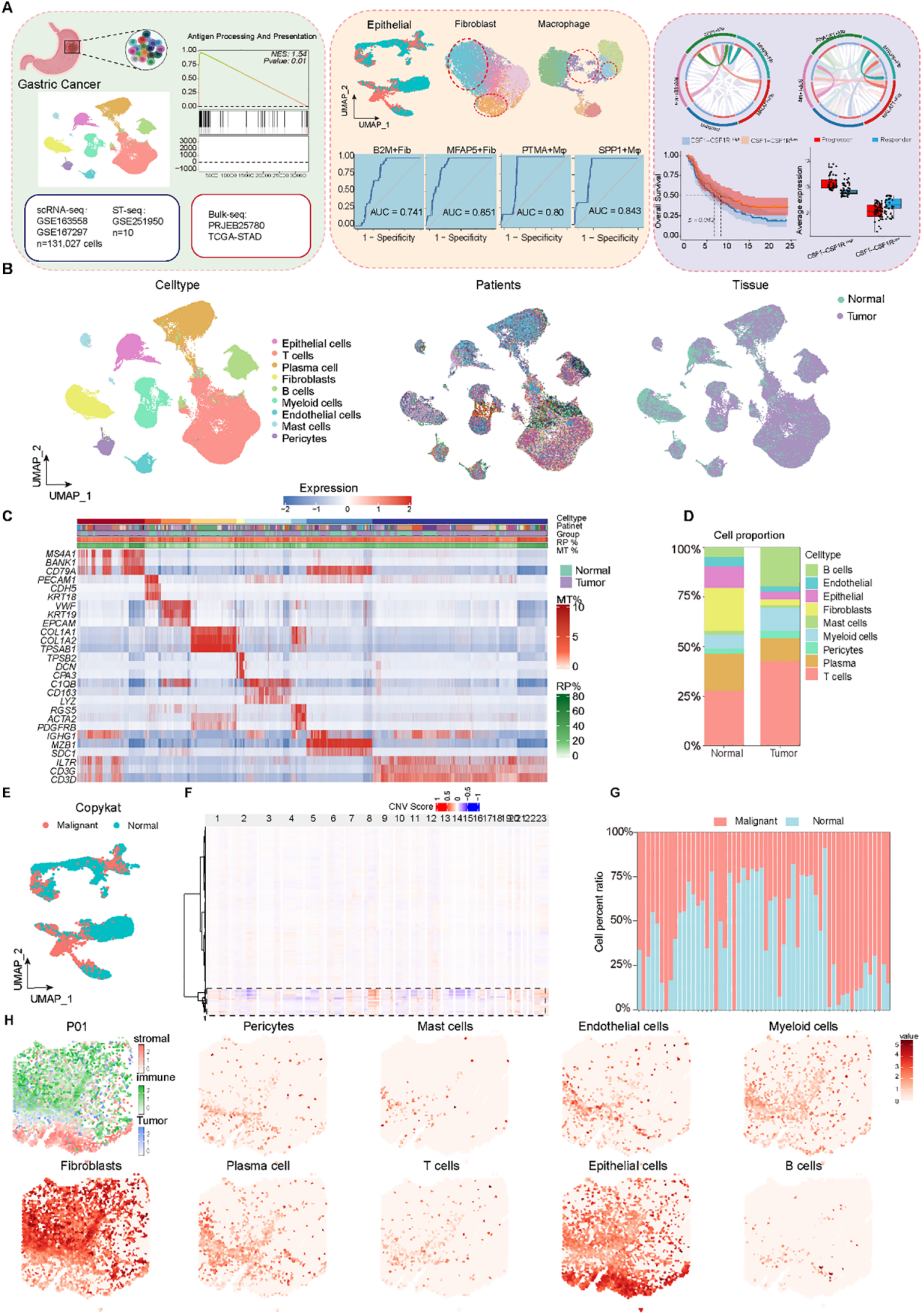
Single-cell and spatial transcriptomic profiling delineates the cellular landscape of gastric cancer. **(a)** Schematic diagram illustrating the workflow of gastric cancer biopsy processing for scRNA-seq analyses. **(b)** UMAP representation of single cells analyzed in this study, colored by major cell type (left), patient identity (middle), and pathological tissue subtype (right). **(c)** Heatmap representation of the top five marker genes across each identified cell type. **(d)** Stacked bar plot depicting the relative abundance of each cell type across distinct pathological subtypes. **(e)** UMAP plot of epithelial cells, colored by CopyKAT-inferred status, distinguishing tumor (aneuploid) and normal (diploid) cells. **(f)** Heatmap of CopyKAT-inferred epithelial cells showing CNV profiles, distinguishing tumors (aneuploid) from normal (diploid) cells. **(g)** Stacked bar plot depicting the relative abundance of normal and tumor epithelial cells across individual patients. **(h)** Feature plot showing the spatial distribution of each cell type. The first panel represents the tumor score (red), stromal score (green), and immune score (blue).

To systematically investigate the cellular composition and spatial organization of the GC tumor microenvironment in relation to immunotherapy resistance, we generated a multiomics atlas by integrating single-cell and spatial transcriptomic profiling. The overall analytical workflow is summarized in the schematic below (**Figure 1A**). To characterize the cellular landscape of gastric cancer comprehensively, we performed scRNA-seq sequencing by integrating multiple previous cohorts (7, 25, 26). After filtering out putative doublets and low-quality cells, a total of 131,027 cells from gastric cancer samples were retained, including 28 normal tissues with 50,268 cells and 34 GC tissues with 80,759 cells (**Figure 1B**). On the basis of the expression of canonical marker genes, the cells were clustered and assigned to 9 major cell types: Pericytes (*RGS5, ACTA2, PDGFRB*), mast cells (*TPSAB1, TPSB2, CPA3*), Endothelial cells (*PECAM1, CDH5, VWF*), Myeloid cells (*LYZ, C1QB, CD163*), B cells (*MS4A1, BANK1, CD79A*), fibroblasts (*COL1A1, COL1A2, DCN*), plasma cells (*IGHG1, MZB1, SDC1*), T cells (*IL7R, CD3G, CD3D*), and Epithelial cells (*KRT18, KRT19, EPCAM*). (**Figure 1C**) (27). Epithelial and myeloid cells constituted the predominant populations, indicating that the TME is shaped by both tumor burden and immune infiltration. (**Figure 1D**). To distinguish malignant from normal epithelium, we performed copy number variation (CNV) analysis via the CopyKAT package, which classifies cells according to their ploidy status (28). This analysis revealed 3,186 aneuploid cells and 5,136 diploid cells in the GC samples. Notably, the aneuploid population was designated as tumor cells. Having established this single-cell atlas, we next sought to resolve the spatial context of these populations. To this end, we performed spatial transcriptomics (ST) on tissue sections from 10 additional GC patients, generating *in situ* gene expression profiles (29). By further integrating single-cell populations with spatial transcriptomic data through deconvolution, we were able to visualize the spatial distributions of distinct cell types across the tissue landscape (**Figure 1H**). Having established this integrated cellular and spatial atlas, we next sought to determine whether these features were associated with the clinical response to immunotherapy.

### 3.2 Gastric cancer transcriptional alterations associated with immunotherapy response and resistance

**Figure 2:**
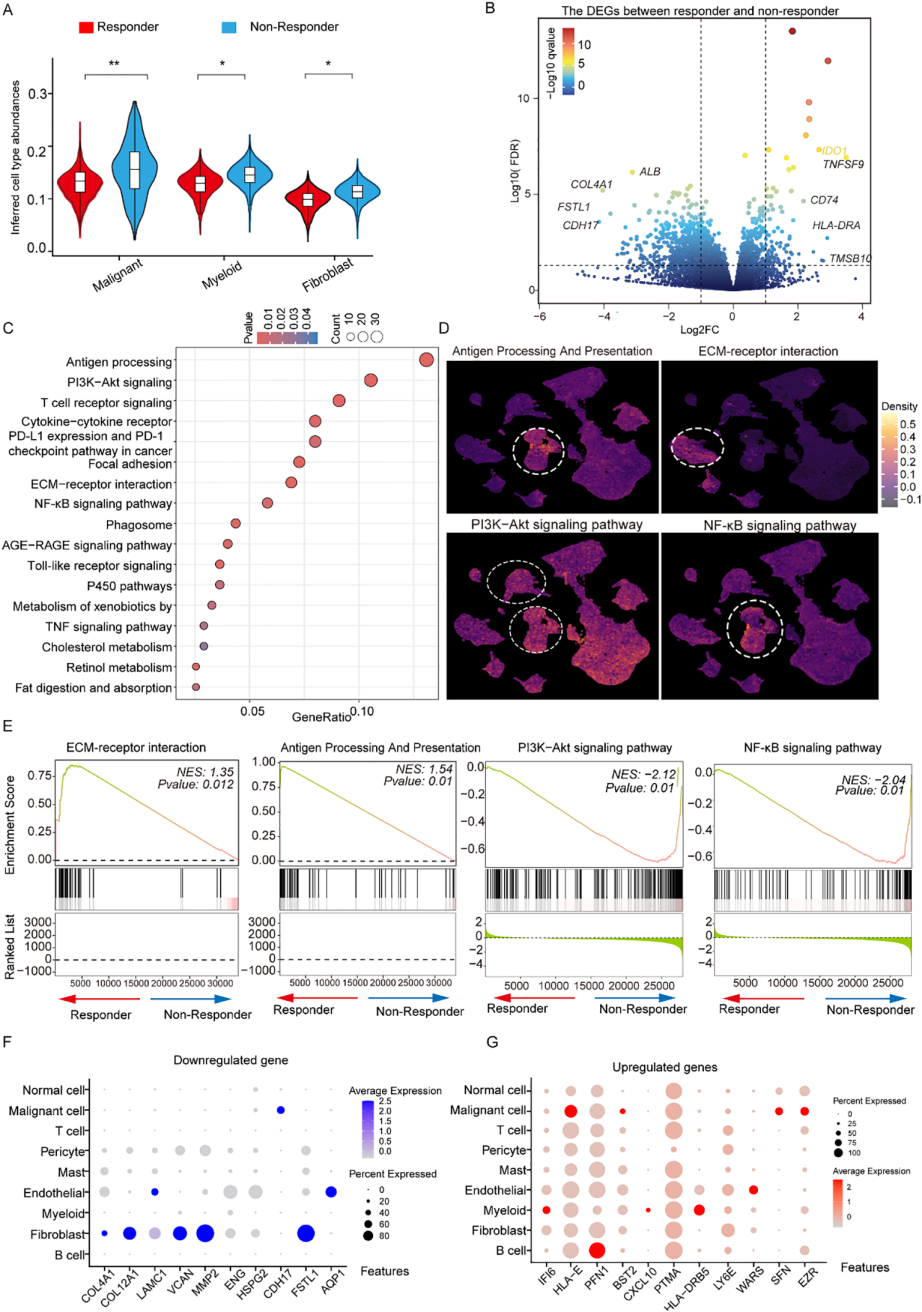
Changes in the transcriptional profiles of different cell types between immunotherapy-resistant and immunotherapy-responsive gastric cancer patients. **(a)** convolution of bulk RNA-seq data via single-cell transcriptomic profiles. The relative proportions of malignant, myeloid, and fibroblast populations were estimated by integrating single-cell and bulk transcriptomic datasets, highlighting the contribution of each major cell type across patient samples. **(b)** Volcano plot illustrating the differential gene expression between immunotherapy-responsive and immunotherapy-resistant gastric cancer patients. **(c)** KEGG pathway enrichment analysis highlighting key signaling pathways associated with immunotherapy resistance and response in gastric cancer. **(d)** Single-cell pathway activity scores for key signaling pathways, including NF-κB, PI3K–Akt, ECM–receptor interaction, and antigen processing and presentation, across different cell types in the gastric cancer single-cell dataset. **(e)** Gene set enrichment analysis (GSEA) of the NF-κB signaling, PI3K–Akt signaling, ECM–receptor interaction, and antigen processing and presentation pathways between immunotherapy progressor and responders with gastric cancer. **(f)** Dot plot illustrating the main cell types contributing to downregulated (blue) gene expression in gastric cancer. **(g)** Dot plot illustrating the main cell types contributing to upregulated (red) gene expression in gastric cancer. *p < 0.05, ** p < 0.01.

To dissect the cellular and molecular underpinnings of immunotherapy resistance, we first leveraged our scRNA-seq atlas as a reference to deconvolve the bulk transcriptomic profiles of an independent immunotherapy cohort (PRJEB25780). This analysis revealed a significant enrichment of malignant cells, myeloid cells, and fibroblasts in progressors compared with responders (**Figure 2A**), suggesting that the expansion of these specific populations is a hallmark of treatment resistance. Subsequently, we performed differential expression and pathway analysis to characterize the divergent molecular landscapes between these two groups (**Figure 2B**). Bulk transcriptomic analysis showed that responders were characterized by the upregulation of genes involved in T-cell activation and interferon-γ signaling (*TNFSF9*, *IDO1*, and *CXCL9*). In contrast, progressors exhibited increased activity in the NF-κB and PI3K-AKT pathways, indicative of a prosurvival and immunosuppressive state (**Figures 2C, D**). Consistent with the deconvolution results, single-cell pathway activity analysis confirmed that malignant cells, myeloid cells, and fibroblasts were the primary contributors to these resistance-associated signaling pathways (**Figure 2E**). Finally, we mapped response-specific gene signatures to our single-cell atlas to pinpoint their cellular origins. Genes upregulated in progressors (*FSTL1* and *COL12A1*) were predominantly expressed by fibroblasts and malignant cells (**Figure 2F**). whereas genes associated with clinical response (*HLA-DRB5* and *CXCL10*) were derived primarily from myeloid cells (**Figure 2G**). Collectively, these findings demonstrate that immunotherapy resistance is underpinned by the coordinated remodeling of the TME, where the expansion of malignant, myeloid, and fibroblast populations establishes an immunosuppressive and treatment-resistant niche.

### 3.3 The tumor-localized MFAP5+fibroblast subset is associated with immunotherapy resistance by shaping an immunosuppressive niche

**Figure 3:**
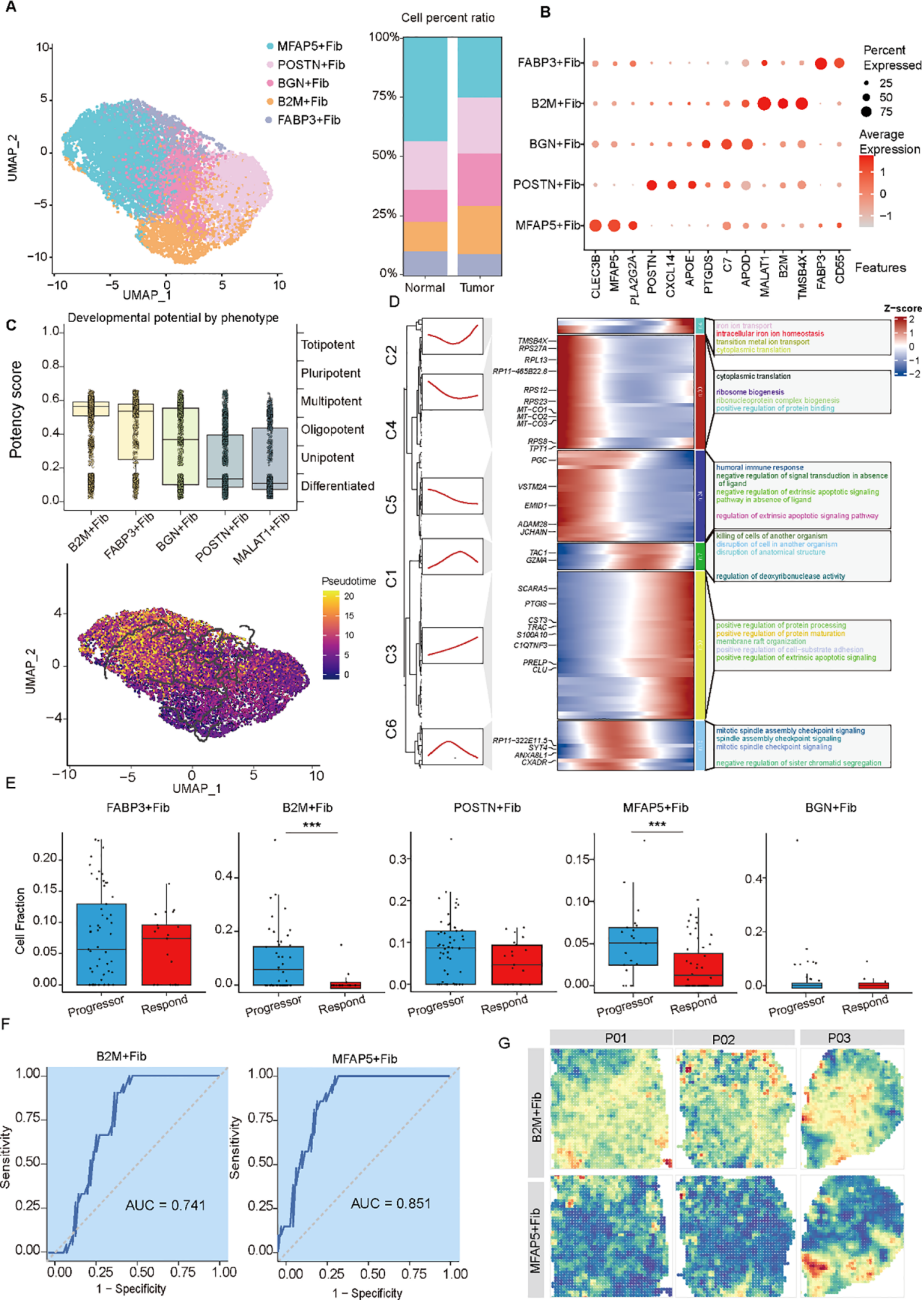
Characterization of fibroblast subpopulations associated with immunotherapy resistance in gastric cancer. **(a)** UMAP plot showing the distribution of fibroblasts in gastric cancer and stacked bar plots depicting the proportions of different fibroblast subtypes in normal and tumor tissues. **(b)** Dot plot of the top 5 marker genes identified for each fibroblast type. **(c)** Boxplot showing CytoTRACE scores across all cell types, representing the differentiation state of each cell population, and expression dynamics of representative genes along the pseudotime trajectory using Monocle3. **(d)** Heatmaps illustrating gene expression patterns and cellular modules associated with pseudotime progression in fibroblasts. **(e)** CIBERSORTx analysis showing fibroblast subtype proportions in responders vs. progressors, highlighting subtypes linked to immune resistance. **(f)** Predictive performance of the B2M+ and MFAP5+ fibroblast subtypes for the immunotherapy response in patients with gastric cancer. **(g)** Spatial distributions of B2M+ and MFAP5+ fibroblasts in the ST dataset, where yellow shading indicates increased expression of the corresponding genes within each spot. *** p < 0.001.

Based on the notable fibroblast enrichment observed in progressors, we hypothesized that distinct fibroblast subsets are responsible for driving tumor progression. We analyzed 11,753 fibroblasts from our scRNA-seq atlas and identified five transcriptionally distinct subtypes (**Figure 3A**). Distinct marker genes for these fibroblast subpopulations were identified (**Figure 3B**). To further investigate their developmental lineage, we performed pseudotime and differentiation potency analyses. The results revealed that MFAP5+ fibroblasts were in an early, high-potency state, suggesting a progenitor-like phenotype (**Figure 3C**). Trajectory analysis revealed that their progression toward functional states was enriched in extracellular matrix (ECM) organization and stroma–tumor interaction pathways (**Figure 3D**). To pinpoint subsets relevant to therapy resistance, we estimated their abundances in the immunotherapy cohort via CIBERSORTx. This analysis revealed that only MFAP5+ and B2M+ fibroblasts were significantly enriched in progressors (**Figure 3E**) and showed predictive value for clinical response (AUC = 0.741 and 0.851, respectively; **Figure 3F**). Spatial transcriptomic mapping revealed a striking anatomical dichotomy: MFAP5+ fibroblasts were predominantly localized within tumor cell nests, whereas B2M+ fibroblasts were restricted to stromal regions (**Figure 3G**). These findings suggest that the MFAP5+ subset is a key mediator of resistance. The tumor-localized distribution, plastic phenotype, and association with ECM remodeling pathways of MFAP5+ fibroblasts suggest that these cells are uniquely positioned to interact directly with malignant cells and sculpt a profibrotic, immunosuppressive niche that hinders an effective immune response (30).

### 3.4 Myeloid cell subpopulations and their associations with the immunotherapy response in gastric cancer

**Figure 4:**
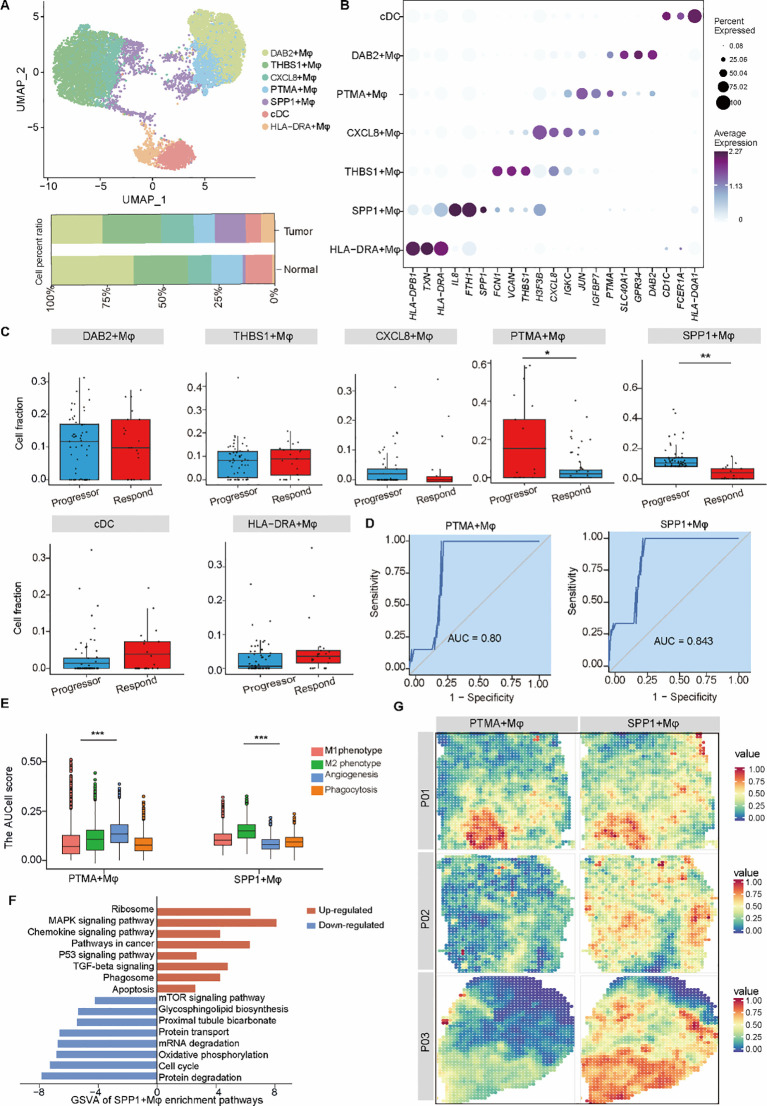
Profiling myeloid cell subtypes linked to immunotherapy resistance in gastric cancer. **(a)** UMAP plot showing the distribution of myeloid cells in gastric cancer and stacked bar plots depicting the proportions of different myeloid cells subtypes in normal and tumor tissues. **(b)** Dot plot of the top 5 marker genes identified for each fibroblast type. **(c)** CIBERSORTx analysis showing fibroblast subtype proportions in responders vs. progressor, highlighting subtypes linked to immune resistance. **(d)** Predictive performance of PTMA+ and SPP1+ macrophages subtypes for immunotherapy response in gastric cancer. **(e)** Evaluation of phagocytosis, angiogenesis, and M1/M2 phenotype pathway activity in the PTMA+ and SPP1+ macrophage subsets. **(f)** GSVA of pathway activity differences in SPP1+ macrophages between normal and tumor-enriched spots. **(g)** Spatial distribution of PTMA+ and SPP1+ macrophages in the ST dataset, where yellow shading indicates increased expression of the corresponding genes within each spot. *p < 0.05, ** p < 0.01.

To characterize the heterogeneity of myeloid cells in gastric cancer, we analyzed a total of 12,591 myeloid cells and identified seven subpopulations: DAB2+ macrophages THBS1+ macrophages, CXCL8+ macrophages, PTMA+ macrophages, SPP1+ macrophages, conventional dendritic cells (cDCs), and HLA-DRA+ macrophages (n = 568) (**Figures 4A, B**). To assess their relevance to immunotherapy outcomes, we applied the CIBERSORTx algorithm to bulk RNA-seq data from treated patients and estimated the proportions of each subtype in responders versus progressors. Among these, PTMA+Mφs and SPP1+Mφs exhibited significant differences between the two groups (**Figure 4C**). Receiver operating characteristic (ROC) analysis further demonstrated moderate predictive accuracy for immunotherapy response, with area under the curve (AUC) values of 0.80 and 0.843 for PTMA+Mφ and SPP1+Mφ, respectively (**Figure 4D**). We next explored the functional states of these two macrophage subsets by evaluating their activities related to phagocytosis, angiogenesis, and macrophage polarization. Both PTMA+Mφs and SPP1+Mφs displayed increased phagocytic and angiogenic potential and exhibited a predominant M2-like phenotype rather than M1-like characteristics (**Figure 4E**). Pathway enrichment analysis revealed that SPP1+ macrophages in tumor tissues were enriched in the TGF-β and chemokine signaling pathways, both of which are associated with immunosuppression and tumor immune evasion (**Figure 4F**). Spatial transcriptomic analysis further demonstrated that PTMA+Mφs and SPP1+Mφs were primarily distributed within tumor regions, suggesting their potential roles in shaping the immunosuppressive tumor microenvironment (**Figure 4G**). Together, these findings highlight that PTMA+ and SPP1+ macrophages exhibit M2-like, protumor properties and preferentially localize within tumor regions, implicating them as key contributors to the establishment of an immunosuppressive microenvironment in gastric cancer.

### 3.5 Spatially resolved immunosuppressive cell–cell interactions in the tumor microenvironment

**Figure 5:**
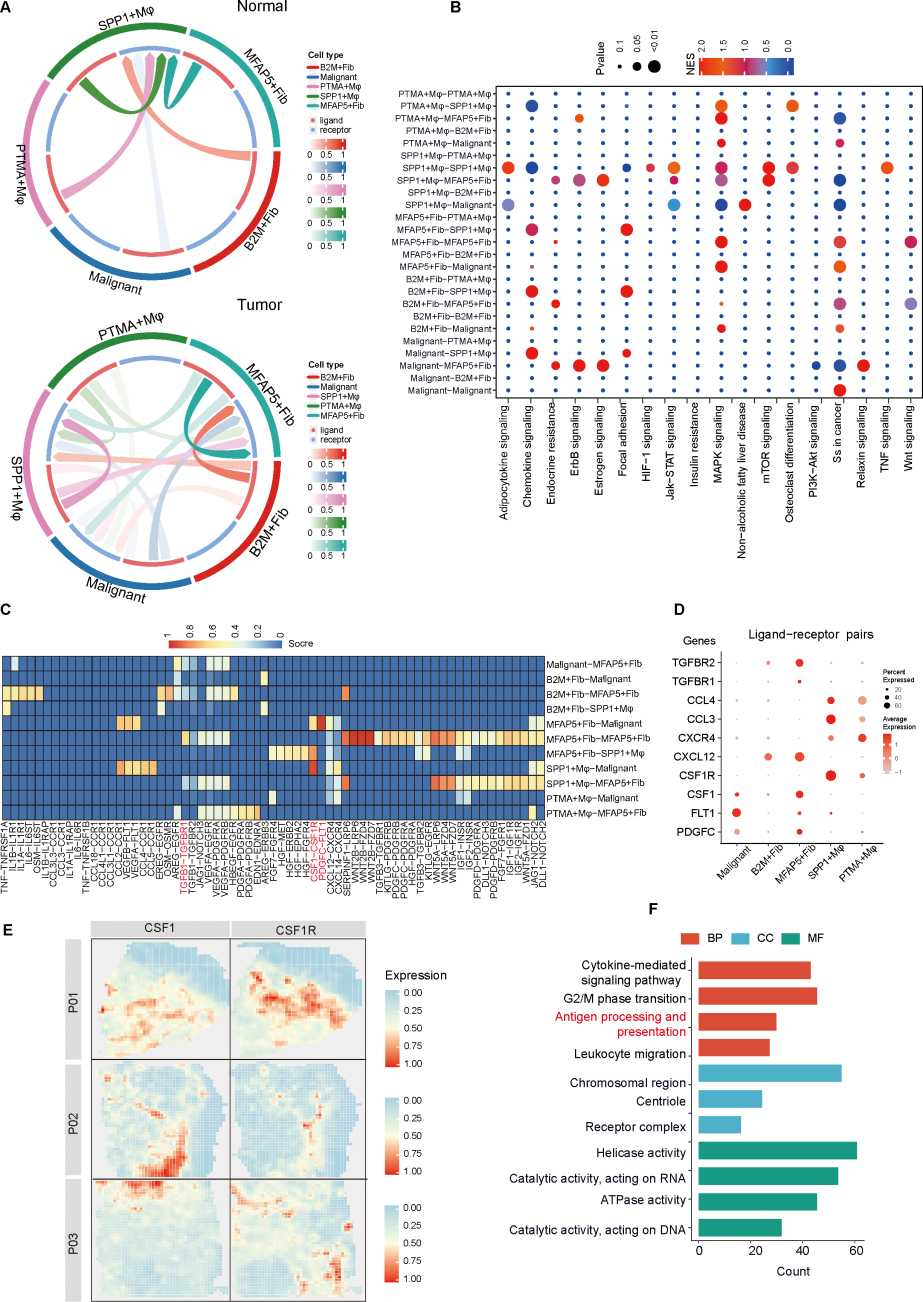
Revealing the tumor microenvironment architecture and intercellular communication in gastric cancer. **(A)** Cell–cell communication analysis via CellCall reveals global interaction patterns between tumor and normal samples in gastric cancer. **(B)** Heatmap showing signaling pathways activated in cell–cell interactions within tumor samples. **(C)** Heatmap showing key receptor–ligand pairs involved in cell–cell interactions. **(D)** Dot plot showing the main cell types expressing key receptor–ligand pairs in the gastric cancer TME. **(E)** Spatial mapping of key receptor–ligand pairs across gastric cancer tissue sections. **(F)** Key receptor–ligand interactions activate pathways associated with immune-therapy resistance.

To further characterize these immune-related cell types and their contributions to the formation of an immunosuppressive microenvironment, we constructed cell–cell communication networks for GCs. Compared with adjacent normal tissues, tumor samples exhibited markedly increased intercellular interactions, suggesting enhanced cellular crosstalk within the tumor microenvironment (**Figure 5A**). To elucidate the biological consequences of these interactions, pathway enrichment analysis revealed significant enrichment of the Wnt, TNF, PI3K–AKT, mTOR, MAPK, JAK–STAT, HIF-1, and chemokine signaling pathways. These pathways are known to mediate immune suppression and tumor-promoting inflammation by driving immune evasion, dysregulated cytokine production, and immune cell dysfunction, thereby fostering an immunosuppressive TME (**Figure 5B**). Among the identified receptor–ligand interactions between immune-related cell types, several key pairs, including *TGFB1–TGFBR1/2*, *HGF–MET*, *WNT5A–FZD7*, and CSF1–CSF1R, are closely associated with immunosuppressive signaling. These interactions promote macrophage polarization, T-cell suppression, and the recruitment of tumor-associated macrophages, collectively contributing to the establishment of an immunosuppressive niche (**Figures 5C, D**). Notably, the CSF1–CSF1R interaction derived from SPP1+Mφ–malignant cell communication clearly exhibited spatial colocalization in tissue sections (**Figure 5E**). Comparative pathway analysis demonstrated that these interacting pairs were enriched in immunosuppressive and protumor signaling pathways, including the TGF-β, PI3K–Akt, and MAPK pathways. Moreover, enrichment of leukocyte migration and cytokine-mediated signaling pathways suggested that these interactions may regulate immune cell trafficking and cytokine exchange, thereby promoting immune evasion, chronic inflammation, and maintenance of an immunosuppressive tumor niche (**Figure 5F**). In summary, enhanced cell–cell interactions in tumors, particularly those involving immunosuppressive receptor–ligand pairs and spatially colocalize and activate pathways that promote immune evasion.

### 3.6 Spatial crosstalk between the CSF1+ epithelium and SPP1+ macrophages shapes an immunosuppressive niche

**Figure 6:**
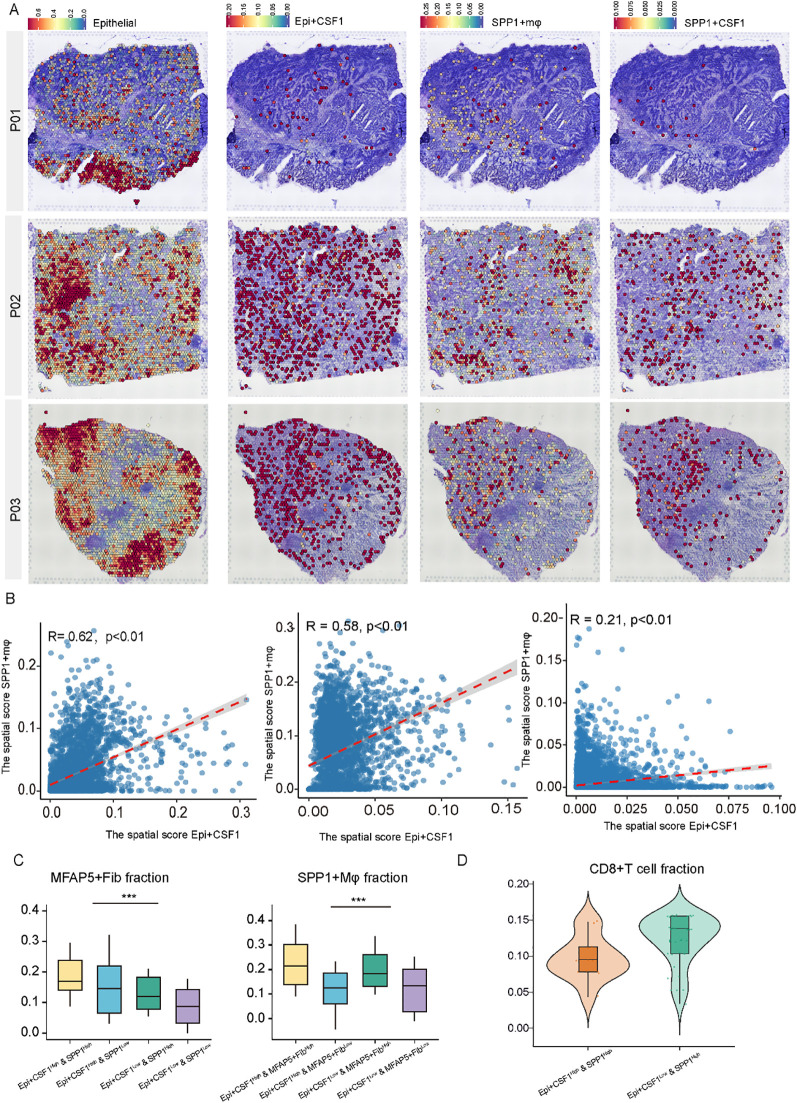
Role of CSF1 in the spatial distribution and cellular interactions in gastric cancer. **(A)** Spatial transcriptomic projection of epithelial cells, CSF1+ epithelial cells, SPP1+ macrophages, and CSF1+SPP1+ macrophages. The spatial distribution of these cell types is shown in tissue sections, with specific markers for each group. Representative samples are included. **(B)** Correlations between tumor cells and SPP1+ macrophage scores inferred from spatial transcriptomics–based cell type deconvolution. **(C)** Deconvolution results of bulk transcriptomics data showing the proportion of MFAP5+ fibroblasts (Fib) in different CSF1+ epithelial cell and SPP1+ macrophage populations, as well as the proportion of SPP1+ macrophages in different CSF1+ epithelial cell and MFAP5+ fibroblast populations. **(D)** Distribution of CD8+ T-cell infiltration within CSF1+ epithelial cells, SPP1+ macrophages, and CSF1+SPP1+ macrophages, as determined via bulk RNA-seq deconvolution analysis. The proportion of CD8+ T cells within each of the specified cell populations is shown. *** p < 0.001.

To comprehensively elucidate the role of the CSF1–CSF1R axis in GC progression, we investigated the spatial organization and intercellular interactions within the TME. Spatial colocalization analysis (**Figure 6A**) revealed a significant association between CSF1+ epithelial cells and SPP1+ macrophages, supported by strong positive correlation coefficients (R = 0.62 p<0.01 and R = 0.58, p< 0.01) (**Figure 6B**). Expanding on this, bulk RNA-seq deconvolution (**Figure 6C**) uncovered a coordinated multicellular ecosystem: the abundance of both CSF1+epithelial cells and SPP1+ macrophages positively correlated with a higher proportion of MFAP5+ fibroblasts. Notably, SPP1+ macrophages were particularly elevated in samples with high infiltration of the other two cell types, suggesting a tripartite synergistic niche. Furthermore, this specific cellular architecture appears to drive immune evasion; deconvolution analysis (**Figure 6D**) revealed a significantly lower proportion of CD8+ T cells in regions characterized by high densities of CSF1+ epithelial cells and SPP1+ macrophages. Collectively, these findings underscore the critical role of the CSF1–CSF1R axis in establishing a tumor-promoting niche that orchestrates intricate cellular crosstalk and potentiates an immunosuppressive microenvironment, thereby facilitating GC progression.

### 3.7 Functional role of CSF1 in gastric cancer progression and its potential as a therapeutic target.

**Figure 7:**
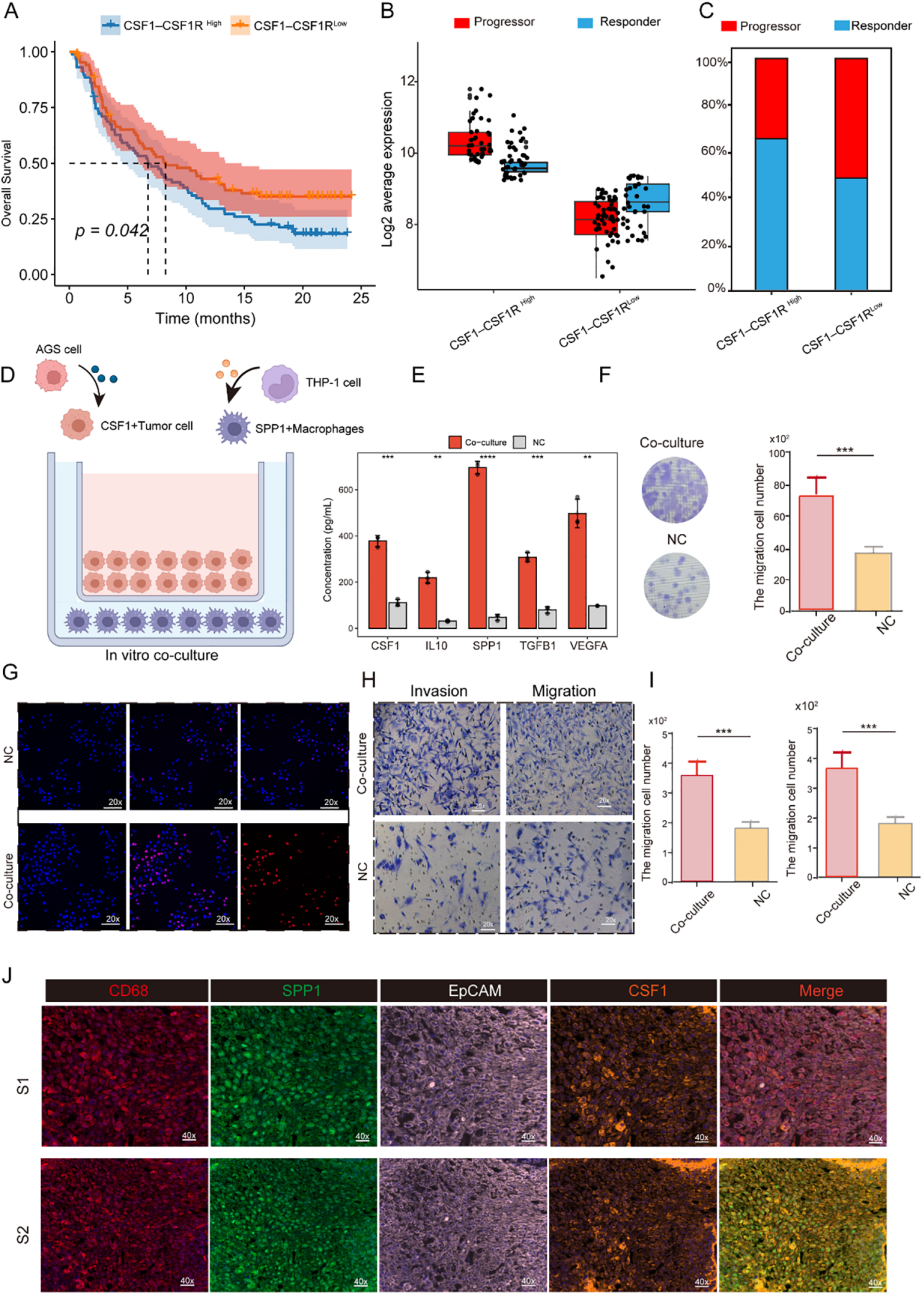
CSF1–CSF1R signaling mediates tumor–macrophage crosstalk to drive progression and immunotherapy resistance. **(A)** Kaplan–Meier curves showing the overall survival **(OS)** of patients in the anti–PD-L1 treatment cohort (IMvigor210), stratified by the CSF1–CSF1R risk score (p = 0.042, log-rank test). **(B)** Comparison of CSF1–CSF1R risk scores between clinical responders (CR/PR) and non-responders (SD/PD) in the IMvigor210 cohort. **(C)** Distribution of clinical responses across low- and high-risk groups. **(D)** Schematic illustration of the non-contact co-culture system using AGS cells and THP-1-derived macrophages. **(E)** ELISA analysis of CSF1, SPP1, and immunosuppressive cytokines (*IL-10*, *TGF-β*, and *VEGFA*) in the supernatants of NC (AGS alone) and Co-culture groups. **(F)** Representative images of the colony formation assay for AGS cells in the NC and Co-culture groups. **(G)** Representative images and quantification of EdU incorporation assays. **(H)** Representative images of Transwell migration and Matrigel invasion assays for AGS cells under the indicated conditions. **(I)** Quantitative analysis of the migration and invasion cell numbers under the indicated conditions. **(J)** Representative multiplex immunofluorescence (mIF) images of human gastric cancer tissues showing the spatial distribution of EPCAM, CD68, CSF1, and SPP1. Nuclei were counterstained with DAPI. *p < 0.05, ** p < 0.01, *** p < 0.001, **** p < 0.0001.

To circumvent the scarcity of GC-specific immunotherapy cohorts, we utilized the IMvigor210 dataset to extrapolate the functional impact of the CSF1–CSF1R axis. In this pan-cancer setting, patients with elevated risk scores exhibited reduced overall survival, underscoring the likely importance of this axis in immune evasion (p = 0.042; **Figure 7A**) and a markedly higher frequency of treatment resistance, with elevated expression observed in progressors compared to clinical responders (**Figures 7B, C**). This clinical correlation prompted us to investigate the paracrine effects of CSF1 within the TME. Using an *in vitro* Transwell co-culture model involving AGS cells and THP-1-derived macrophages, we observed a marked synergistic upregulation in the secretion of CSF1 and SPP1, accompanied by the robust induction of immunosuppressive cytokines, such as *IL-10*, *TGF-β1*, and *VEGFA* (**Figures 7D, E**). This secretomic remodeling significantly fueled tumor progression; compared to the NC group, AGS cells in the co-culture system exhibited enhanced long-term proliferative potential and self-renewal capacity, as evidenced by increased colony formation (**Figures 7F, G**). Furthermore, the co-culture environment potentiated their migratory and invasive properties (**Figures 7H, I**). Finally, multiplex immunofluorescence (mIF) confirmed the spatial proximity and co-localization of CSF1+ tumor cells and SPP1+macrophages in clinical specimens (**Figure 7J**), providing *in situ* evidence that this signaling axis orchestrates a pro-tumorigenic niche. Collectively, these findings demonstrate that CSF1 promotes gastric cancer progression not only through intrinsic signaling but also by shaping a macrophage-dependent immunosuppressive milieu.

## 4 Discussion

GC exhibits substantial cellular and molecular heterogeneity, driving tumor progression and therapeutic resistance (8, 27, 31). By integrating scRNA-seq, spatial transcriptomics, and bulk RNA-seq, we characterized the GC TME to investigate the mechanisms underlying immune resistance and their impact on the immunotherapy response.

All nine major cell types were identified, with epithelial and myeloid cells predominating. CNV analysis distinguished aneuploid tumor epithelial cells from diploid nonmalignant cells. Integration with immunotherapy data revealed enrichment of malignant, fibroblast, and myeloid cells in progressors, suggesting their role in immune evasion. Fibroblast subclustering revealed five subtypes, with MFAP5+ and B2M+ fibroblasts showing high differentiation potential and associations with the immunotherapy response. Pseudotime analysis revealed a progression from metabolic and translational states to immune-regulatory, ECM remodeling, and proliferative programs. Spatial mapping revealed that MFAP5+ fibroblasts were enriched in tumor regions, whereas B2M+ fibroblasts were localized to stromal areas. The myeloid subpopulations included seven macrophage and dendritic cell subsets, with PTMA+ and SPP1+ macrophages enriched in progressor. Functional scoring indicated enhanced angiogenic and phagocytic activity and a predominant M2-like phenotype. TGF-β and chemokine signaling were enriched, supporting immunosuppression, and these macrophages colocalized within tumor regions (32).

Cell–cell communication analysis revealed increased intercellular interactions in tumors, with immunosuppressive receptor–ligand pairs *CSF1–CSF1R*, *TGFB1–TGFBR1/2* and, *WNT5A–FZD7* promoting immuno-suppression of T cells, macrophage polarization, and recruitment of tumor-associated macrophages (33–35). The MFAP5+ fibroblasts and malignant cells strongly colocalized, indicating that fibroblast–malignant crosstalk is involved in immune evasion.

Functional validation confirmed that CSF1, which is highly expressed in GC tissues and cell lines, promotes tumor proliferation, migration, and invasion, suggesting that targeting *CSF1–CSF1R* signaling could disrupt stromal–tumor crosstalk and attenuate macrophage-mediated immunosuppression.

Our study has several limitations. First, the sample size for single-cell and spatial transcriptomic profiling was relatively modest, which may limit the generalizability of our findings. Second, functional validation relies primarily on *in vitro* cell line models, which do not fully recapitulate the complexity of the TME. Third, although spatial transcriptomics provides valuable insights into cellular colocalization, the resolution of current platforms may not capture all fine-scale interactions between cells. Finally, while our integrative analysis highlighted key receptor-ligand pairs and immunosuppressive pathways, further *in vivo* studies are needed to validate these targets and evaluate combinatorial therapeutic strategies targeting both malignant and stromal compartments.

In summary, our integrative analysis revealed the immunosuppressive microenvironment in GC that is mediated by malignant-associated cellular communication. CSF1–CSF1R signaling has emerged as a potential axis driving tumor progression and immune evasion, highlighting promising avenues for targeted therapeutic intervention and precision immunotherapy.

## Data Availability

The public datasets utilized in this study can be accessed via the following repositories: scRNA-seq profiles of human gastric cancer are available in GEO under GSE167297 and GSE163558; spatial transcriptomics data are hosted under GEO accession GSE251950; bulk RNA-seq cohorts are accessible through ENA (PRJEB25780) and the TCGA Data Portal (TCGA-STAD). Pre-processing, including quality control and normalization of raw expression matrices, was performed following the standardized pipelines established in the original cohorts.

## References

[1] Siegel RL Miller KD Fuchs HE Jemal A . Cancer statistics, 2022. CA Cancer J Clin. (2022) 72:7–33. doi: 10.3322/caac.21708 35020204

[2] Smyth EC Nilsson M Grabsch HI van Grieken NC Lordick F . Gastric cancer. Lancet. (2020) 396:635–48. doi: 10.1016/s0140-6736(20)31288-5 32861308

[3] Correa P Haenszel W Cuello C Tannenbaum S Archer M . A model for gastric cancer epidemiology. Lancet. (1975) 2:58–60. doi: 10.1016/s0140-6736(75)90498-5 49653

[4] Janjigian YY Kawazoe A Yañez P Li N Lonardi S Kolesnik O . The keynote-811 trial of dual Pd-1 and Her2 blockade in Her2-positive gastric cancer. Nature. (2021) 600:727–30. doi: 10.1038/s41586-021-04161-3 34912120 PMC8959470

[5] Janjigian YY Shitara K Moehler M Garrido M Salman P Shen L . First-line nivolumab plus chemotherapy versus chemotherapy alone for advanced gastric, gastro-oesophageal junction, and oesophageal adenocarcinoma (Checkmate 649): a randomised, open-label, phase 3 trial. Lancet. (2021) 398:27–40. doi: 10.1016/s0140-6736(21)00797-2 34102137 PMC8436782

[6] Song J Wei R Liu C Zhao Z Liu X Wang Y . Antigen-presenting cancer associated fibroblasts enhance antitumor immunity and predict immunotherapy response. Nat Commun. (2025) 16:2175. doi: 10.1038/s41467-025-57465-7 40038297 PMC11880398

[7] Stuart T Butler A Hoffman P Hafemeister C Papalexi E Mauck WM . Comprehensive integration of single-cell data. Cell. (2019) 177:1888–1902.e21. doi: 10.1016/j.cell.2019.05.031 31178118 PMC6687398

[8] Cristescu R Lee J Nebozhyn M Kim KM Ting JC Wong SS . Molecular analysis of gastric cancer identifies subtypes associated with distinct clinical outcomes. Nat Med. (2015) 21:449–56. doi: 10.1038/nm.3850 25894828

[9] Chang Z Zhong C Xu S Zhang Y Guo X Yu J . Multi-modal integration of histopathology and transcriptomics reveals Stab1(+) macrophage-associated efferocytosis as a suppressive immune mechanism in colon adenocarcinoma. J Transl Med. (2025) 23:1386. doi: 10.1186/s12967-025-07348-8 41361444 PMC12683853

[10] Sathe A Grimes SM Lau BT Chen J Suarez C Huang RJ . Single-cell genomic characterization reveals the cellular reprogramming of the gastric tumor microenvironment. Clin Cancer Res. (2020) 26:2640–53. doi: 10.1158/1078-0432.Ccr-19-3231 32060101 PMC7269843

[11] Zhang M Hu S Min M Ni Y Lu Z Sun X . Dissecting transcriptional heterogeneity in primary gastric adenocarcinoma by single cell Rna sequencing. Gut. (2021) 70:464–75. doi: 10.1136/gutjnl-2019-320368 32532891 PMC7873416

[12] Zhang G Zhang X Pan W Chen X Wan L Liu C . Dissecting the spatial and single-cell transcriptomic architecture of cancer stem cell niche driving tumor progression in gastric cancer. Adv Sci (Weinh). (2025) 12:e2413019. doi: 10.1002/advs.202413019 39950944 PMC12079437

[13] Lewis SM Asselin-Labat M-L Nguyen Q Berthelet J Tan X Wimmer VC . Spatial omics and multiplexed imaging to explore cancer biology. Nat Methods. (2021) 18:997–1012. doi: 10.1038/s41592-021-01203-6 34341583

[14] Zhang P Yang M Zhang Y Xiao S Lai X Tan A . Dissecting the single-cell transcriptome network underlying gastric premalignant lesions and early gastric cancer. Cell Rep. (2019) 27:1934–1947.e5. doi: 10.1016/j.celrep.2019.04.052 31067475

[15] Kumar V Ramnarayanan K Sundar R Padmanabhan N Srivastava S Koiwa M . Single-cell atlas of lineage states, tumor microenvironment, and subtype-specific expression programs in gastric cancer. Cancer Discov. (2022) 12:670–91. doi: 10.1158/2159-8290.Cd-21-0683 34642171 PMC9394383

[16] Hao Y Hao S Andersen-Nissen E Mauck WM Zheng S . Integrated analysis of multimodal single-cell data. Cell. (2021) 184:3573–3587.e29. doi: 10.1016/j.cell.2021.04.048 34062119 PMC8238499

[17] Dobin A Davis CA Schlesinger F Drenkow J Zaleski C Jha S . Star: ultrafast universal Rna-seq aligner. Bioinformatics. (2013) 29:15–21. doi: 10.1093/bioinformatics/bts635 23104886 PMC3530905

[18] Korsunsky I Millard N Fan J Slowikowski K Zhang F Wei K . Fast, sensitive and accurate integration of single-cell data with Harmony. Nat Methods. (2019) 16:1289–96. doi: 10.1038/s41592-019-0619-0 31740819 PMC6884693

[19] Love MI Huber W Anders S . Moderated estimation of fold change and dispersion for Rna-seq data with Deseq2. Genome Biol. (2014) 15:550. doi: 10.1186/s13059-014-0550-8 25516281 PMC4302049

[20] Hänzelmann S Castelo R Guinney J . Gsva: gene set variation analysis for microarray and Rna-seq data. BMC Bioinf. (2013) 14:7. doi: 10.1186/1471-2105-14-7 23323831 PMC3618321

[21] Liberzon A Subramanian A Pinchback R Thorvaldsdóttir H Tamayo P Mesirov JP . Molecular signatures database (Msigdb) 3.0. Bioinformatics. (2011) 27:1739–40. doi: 10.1093/bioinformatics/btr260 21546393 PMC3106198

[22] Yu G Wang LG Han Y He QY . Clusterprofiler: an R package for comparing biological themes among gene clusters. Omics. (2012) 16:284–7. doi: 10.1089/omi.2011.0118 22455463 PMC3339379

[23] Qiu X Mao Q Tang Y Wang L Chawla R Pliner HA . Reversed graph embedding resolves complex single-cell trajectories. Nat Methods. (2017) 14:979–82. doi: 10.1038/nmeth.4402 28825705 PMC5764547

[24] Zhang Y Liu T Hu X Wang M Wang J Zou B . Cellcall: integrating paired ligand-receptor and transcription factor activities for cell-cell communication. Nucleic Acids Res. (2021) 49:8520–34. doi: 10.1093/nar/gkab638 34331449 PMC8421219

[25] Kang B Camps J Fan B Jiang H Ibrahim MM Hu X . Parallel single-cell and bulk transcriptome analyses reveal key features of the gastric tumor microenvironment. Genome Biol. (2022) 23:265. doi: 10.1186/s13059-022-02828-2 36550535 PMC9773611

[26] Jeong HY Ham IH Lee SH Ryu D Son SY Han SU . Spatially distinct reprogramming of the tumor microenvironment based on tumor invasion in diffuse-type gastric cancers. Clin Cancer Res. (2021) 27:6529–42. doi: 10.1158/1078-0432.Ccr-21-0792 34385296

[27] Wang R Dang M Harada K Han G Wang F Pool Pizzi M . Single-cell dissection of intratumoral heterogeneity and lineage diversity in metastatic gastric adenocarcinoma. Nat Med. (2021) 27:141–51. doi: 10.1038/s41591-020-1125-8 33398161 PMC8074162

[28] Gao R Bai S Henderson YC Lin Y Schalck A Yan Y . Delineating copy number and clonal substructure in human tumors from single-cell transcriptomes. Nat Biotechnol. (2021) 39:599–608. doi: 10.1038/s41587-020-00795-2 33462507 PMC8122019

[29] Sun C Wang A Zhou Y Chen P Wang X Huang J . Spatially resolved multi-omics highlights cell-specific metabolic remodeling and interactions in gastric cancer. Nat Commun. (2023) 14:2692. doi: 10.1038/s41467-023-38360-5 37164975 PMC10172194

[30] Kieffer Y Hocine HR Gentric G Pelon F Bernard C Bourachot B . Single-cell analysis reveals fibroblast clusters linked to immunotherapy resistance in cancer. Cancer Discov. (2020) 10:1330–51. doi: 10.1158/2159-8290.Cd-19-1384 32434947

[31] Comprehensive molecular characterization of gastric adenocarcinoma. Nature. (2014) 513:202–9. doi: 10.1038/nature13480 25079317 PMC4170219

[32] Ren Q Zhu P Zhang H Ye T Liu D Gong Z . Identification and validation of stromal-tumor microenvironment-based subtypes tightly associated with Pd-1/Pd-L1 immunotherapy and outcomes in patients with gastric cancer. Cancer Cell Int. (2020) 20:92. doi: 10.1186/s12935-020-01173-3 32226313 PMC7092673

[33] Cannarile MA Weisser M Jacob W Jegg AM Ries CH Rüttinger D . Colony-stimulating factor 1 receptor (Csf1r) inhibitors in cancer therapy. J Immunother Cancer. (2017) 5:53. doi: 10.1186/s40425-017-0257-y 28716061 PMC5514481

[34] Huang YH Cai K Xu PP Wang L Huang CX Fang Y . Crebbp/Ep300 mutations promoted tumor progression in diffuse large B-cell lymphoma through altering tumor-associated macrophage polarization via Fbxw7-Notch-Ccl2/Csf1 axis. Signal Transduct Target Ther. (2021) 6:10. doi: 10.1038/s41392-020-00437-8 33431788 PMC7801454

[35] Thierry GR Baudon EM Bijnen M Bellomo A Lagueyrie M Mondor I . Non-classical monocytes scavenge the growth factor Csf1 from endothelial cells in the peripheral vascular tree to ensure survival and homeostasis. Immunity. (2024) 57:2108–2121.e6. doi: 10.1016/j.immuni.2024.07.005 39089257

