# Supplementary figures and images for: The CSF1+ tumor cell–SPP1+ macrophage axis drives gastric cancer progression and immunotherapy resistance
Source: Front Immunol. 2026 Jun 16;17:1817573. doi: 10.3389/fimmu.2026.1817573 (PMC13314632; doi:10.3389/fimmu.2026.1817573)

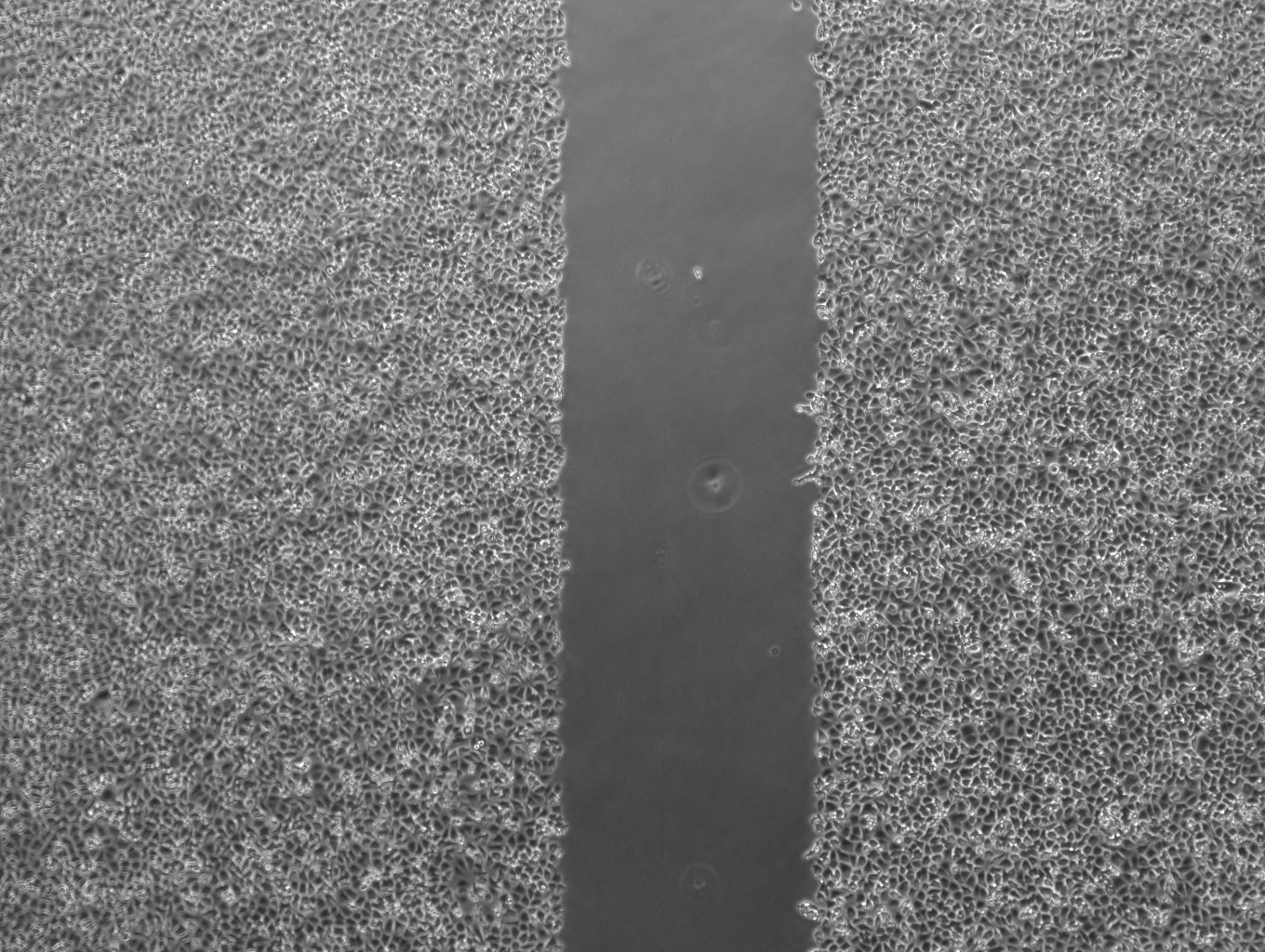

Supplement: Supplementary file 1 [file DataSheet1.zip › 0h/nc-2/图像_65668.tif]

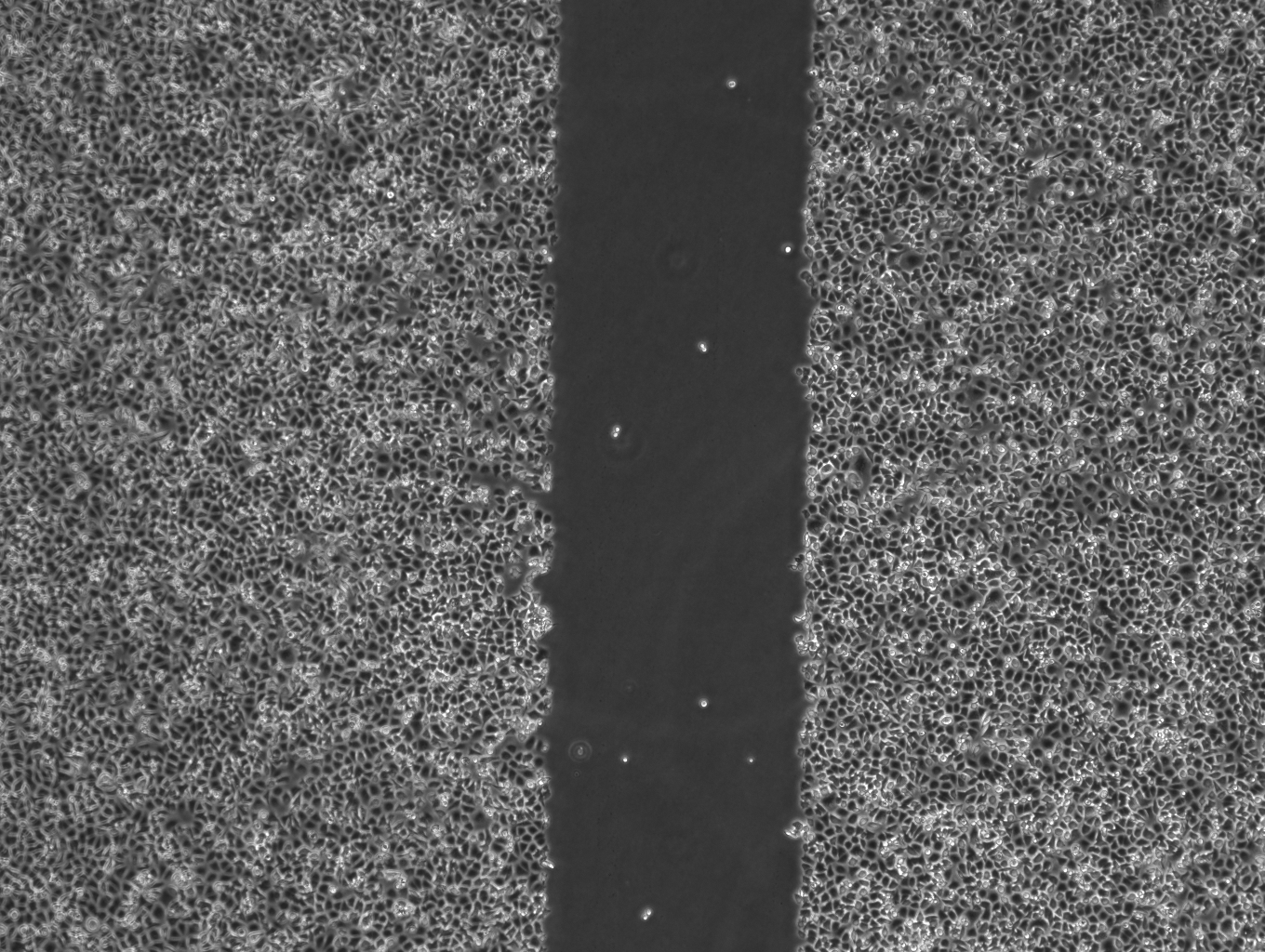

Supplement: Supplementary file 1 [file DataSheet1.zip › 0h/nc-2/图像_65669.tif]

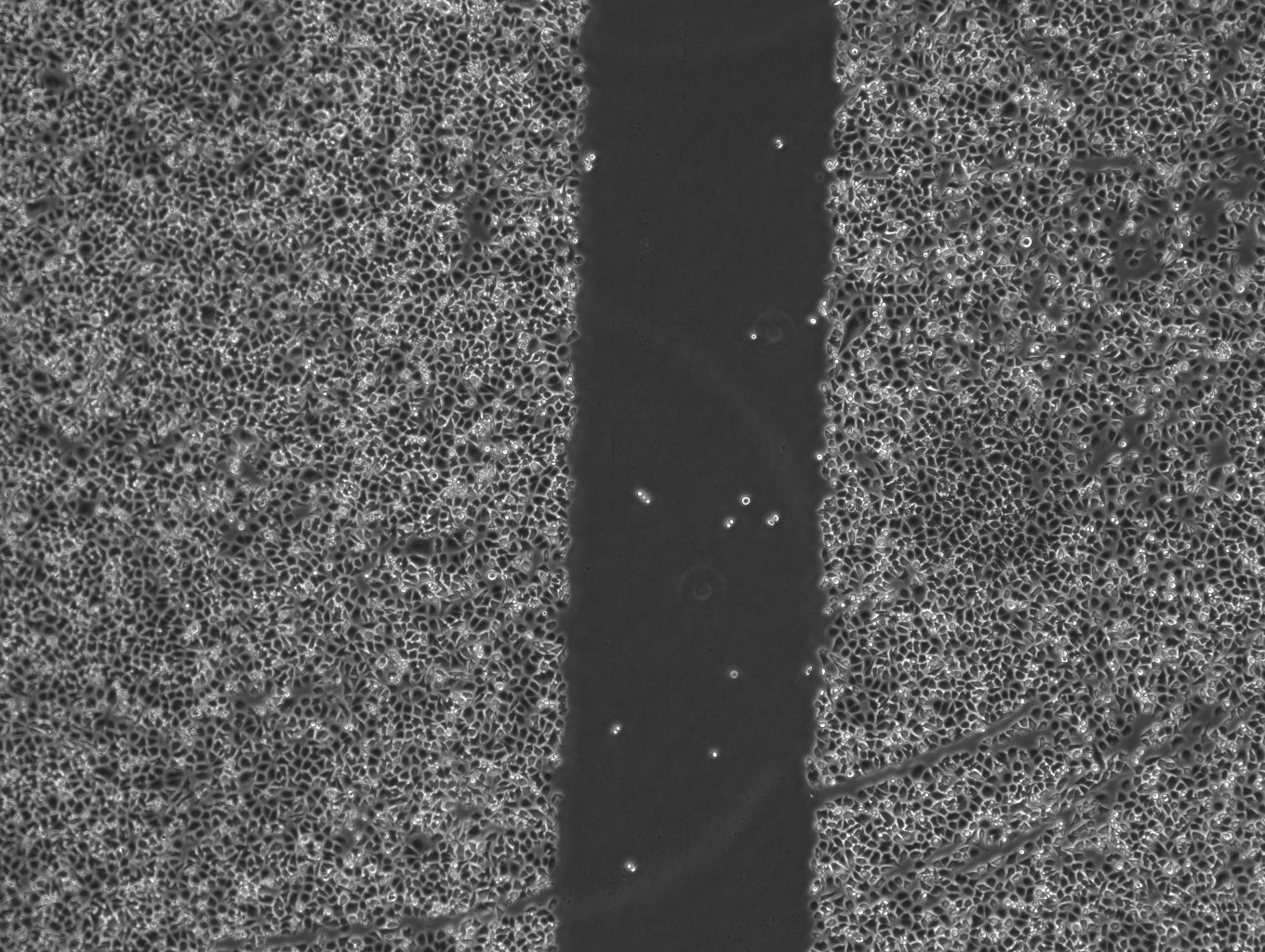

Supplement: Supplementary file 1 [file DataSheet1.zip › 0h/nc-2/图像_65670.tif]

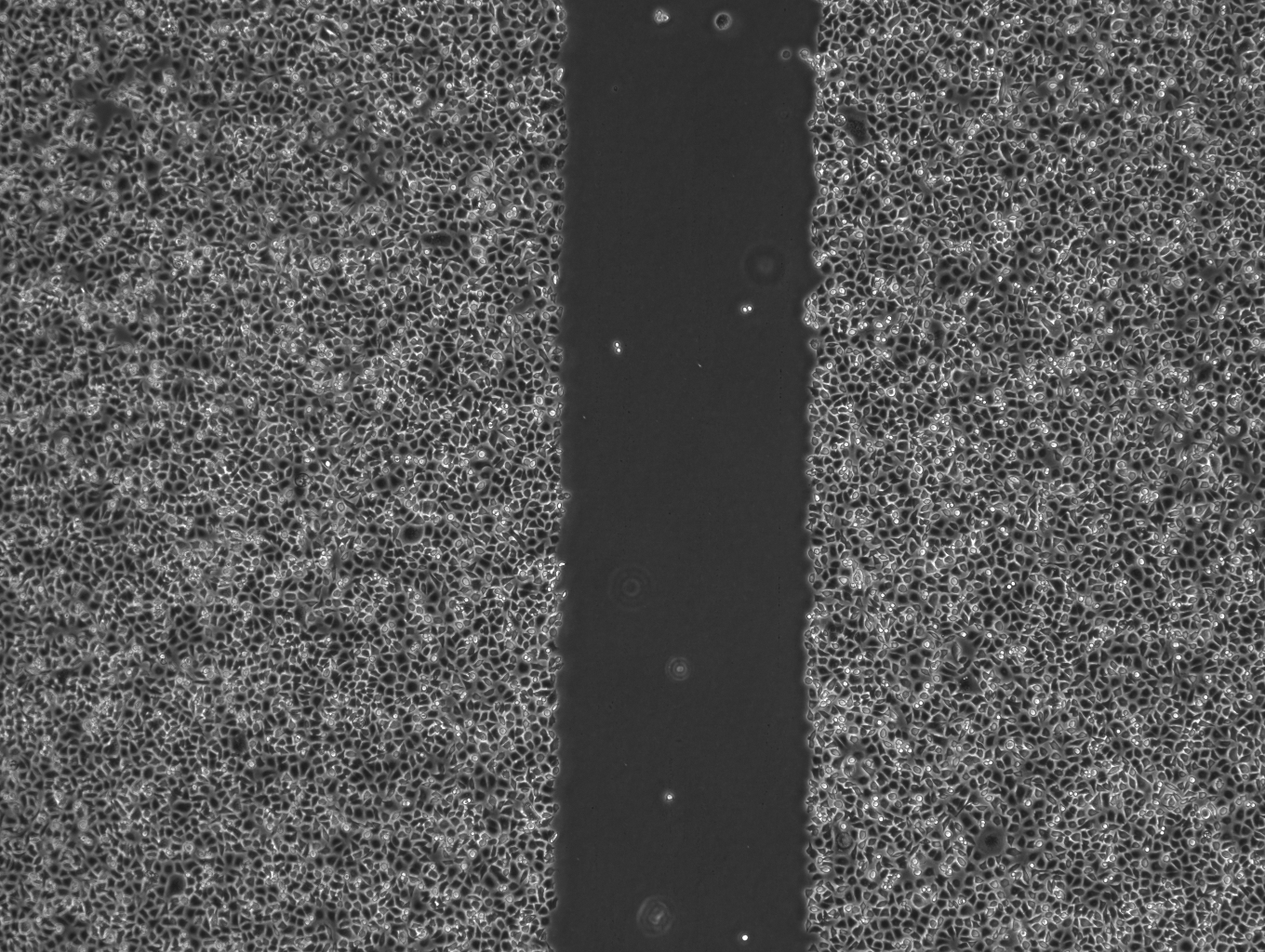

Supplement: Supplementary file 1 [file DataSheet1.zip › 0h/nc-2/图像_65671.tif]

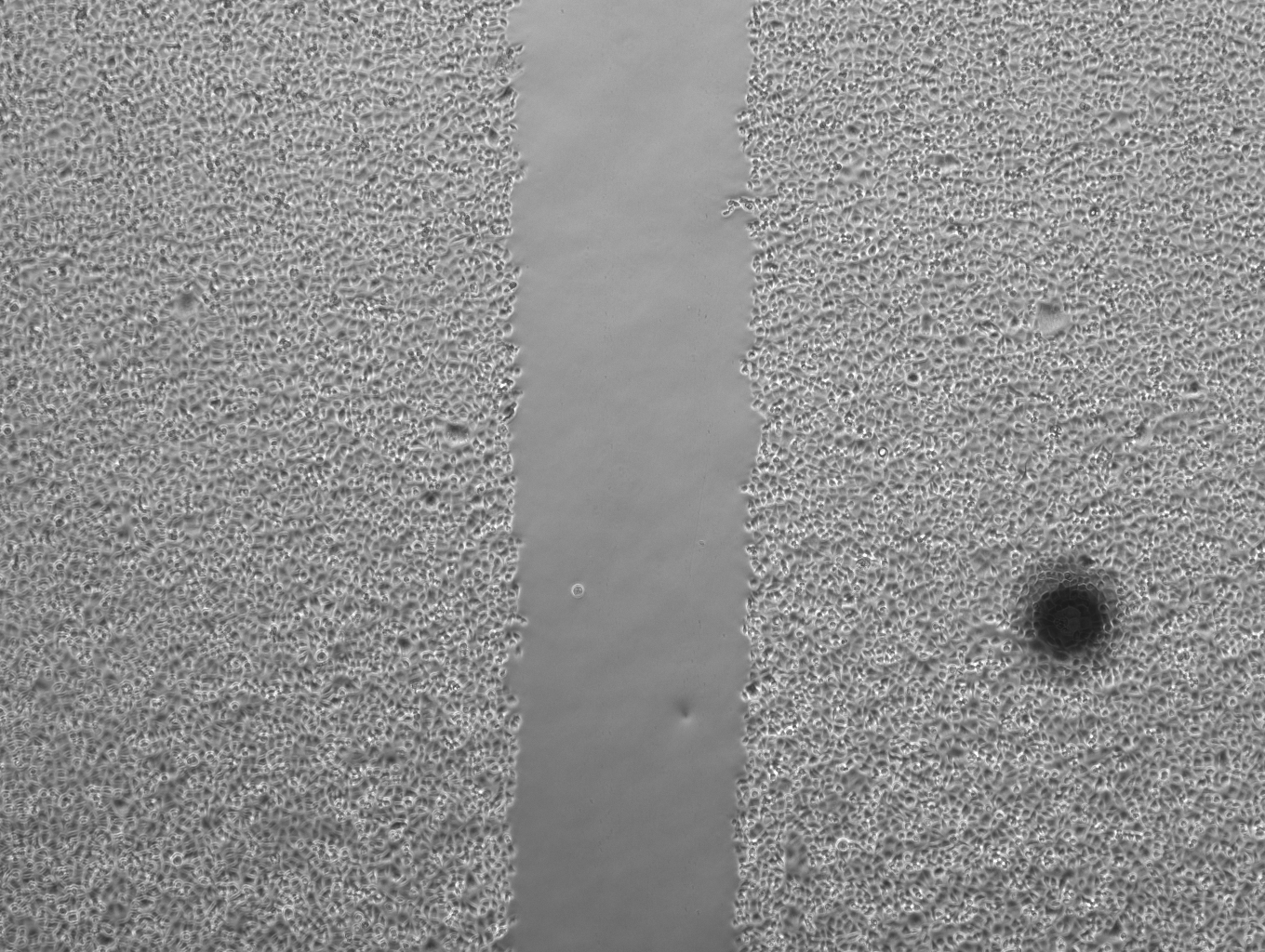

Supplement: Supplementary file 1 [file DataSheet1.zip › 0h/si-1/图像_65680.tif]

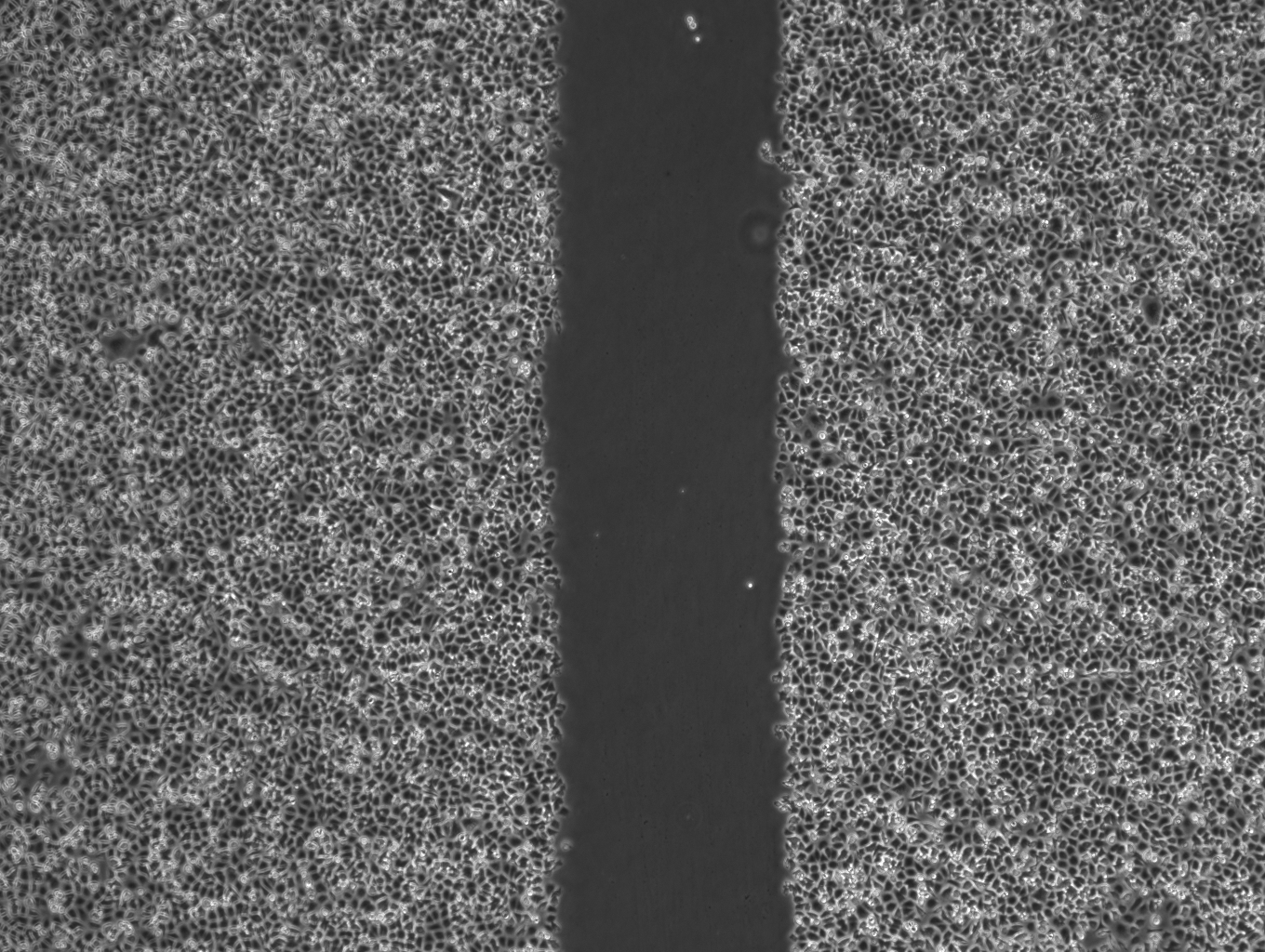

Supplement: Supplementary file 1 [file DataSheet1.zip › 0h/si-1/图像_65681.tif]

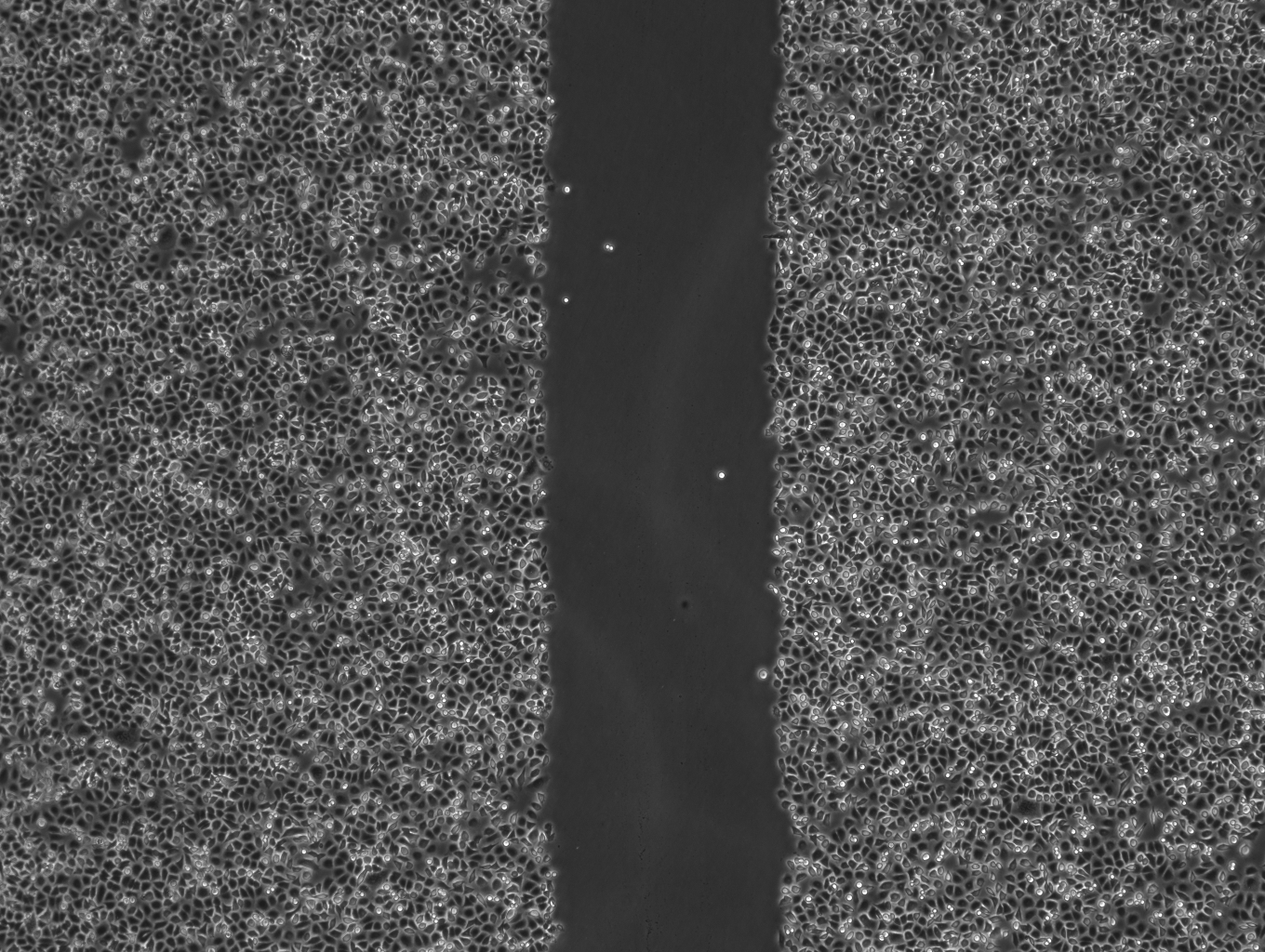

Supplement: Supplementary file 1 [file DataSheet1.zip › 0h/si-1/图像_65682.tif]

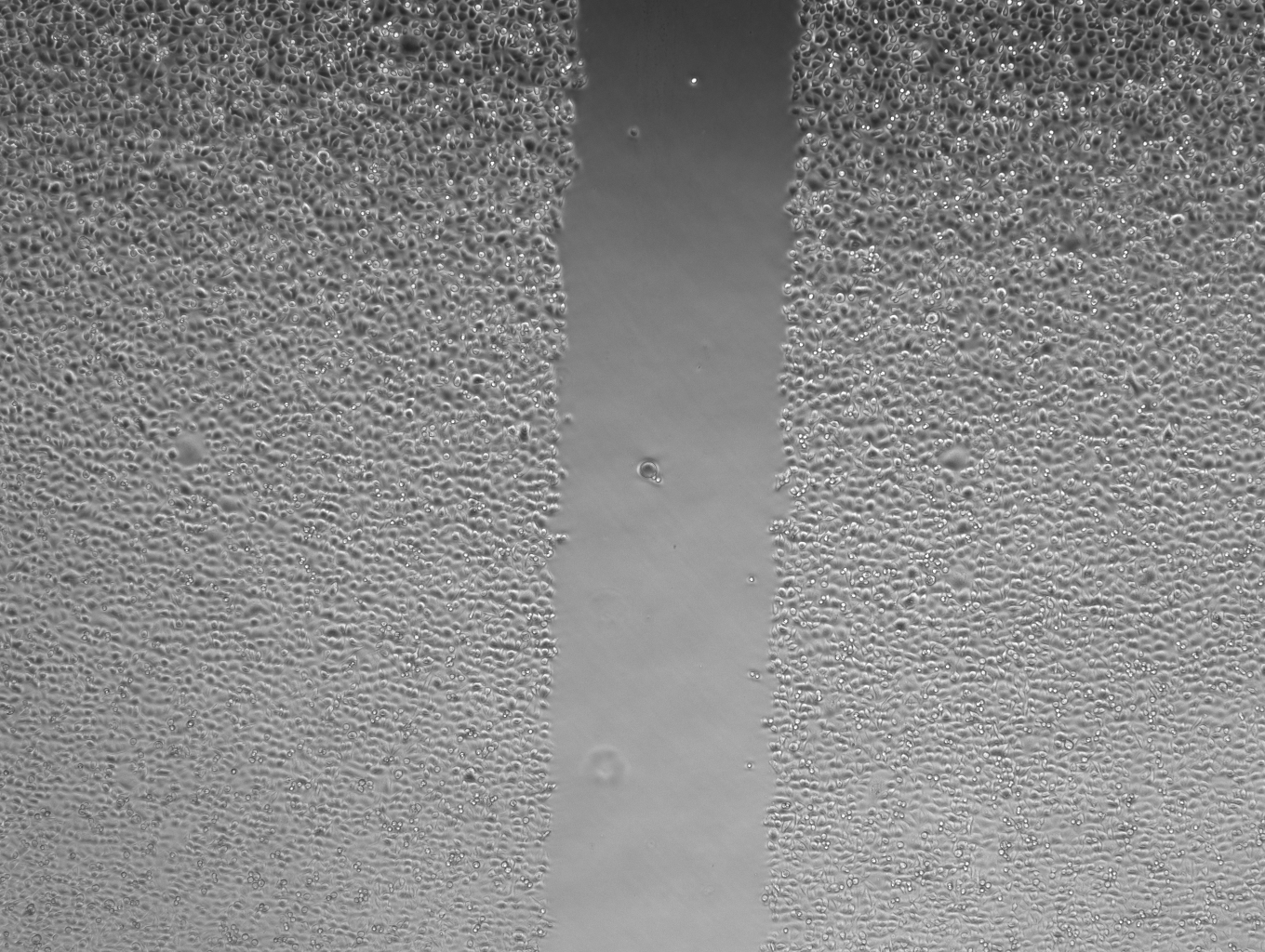

Supplement: Supplementary file 1 [file DataSheet1.zip › 0h/si-1/图像_65683.tif]

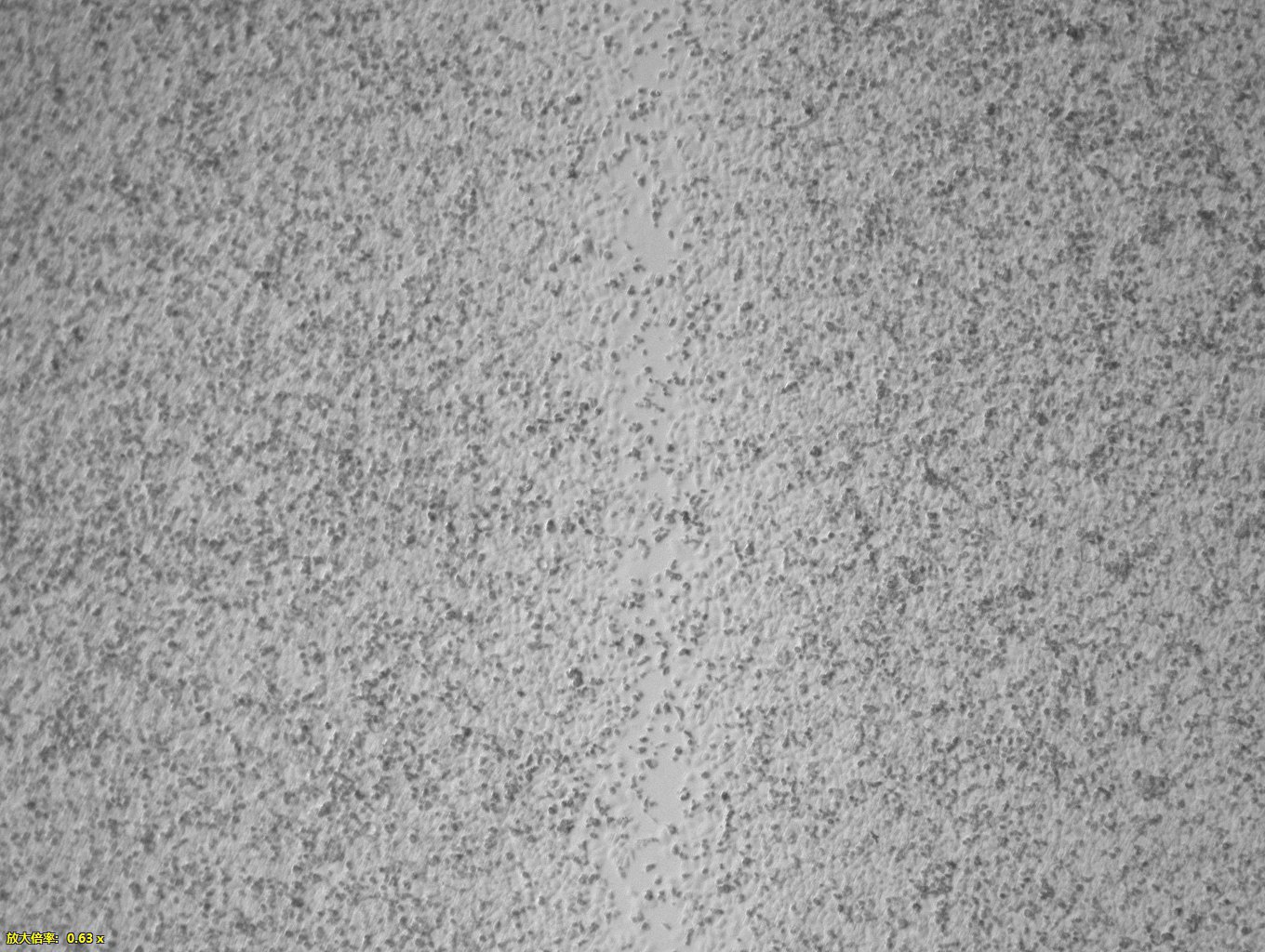

Supplement: Supplementary file 1 [file DataSheet1.zip › 48h/nc-2/图像_66650.jpg]

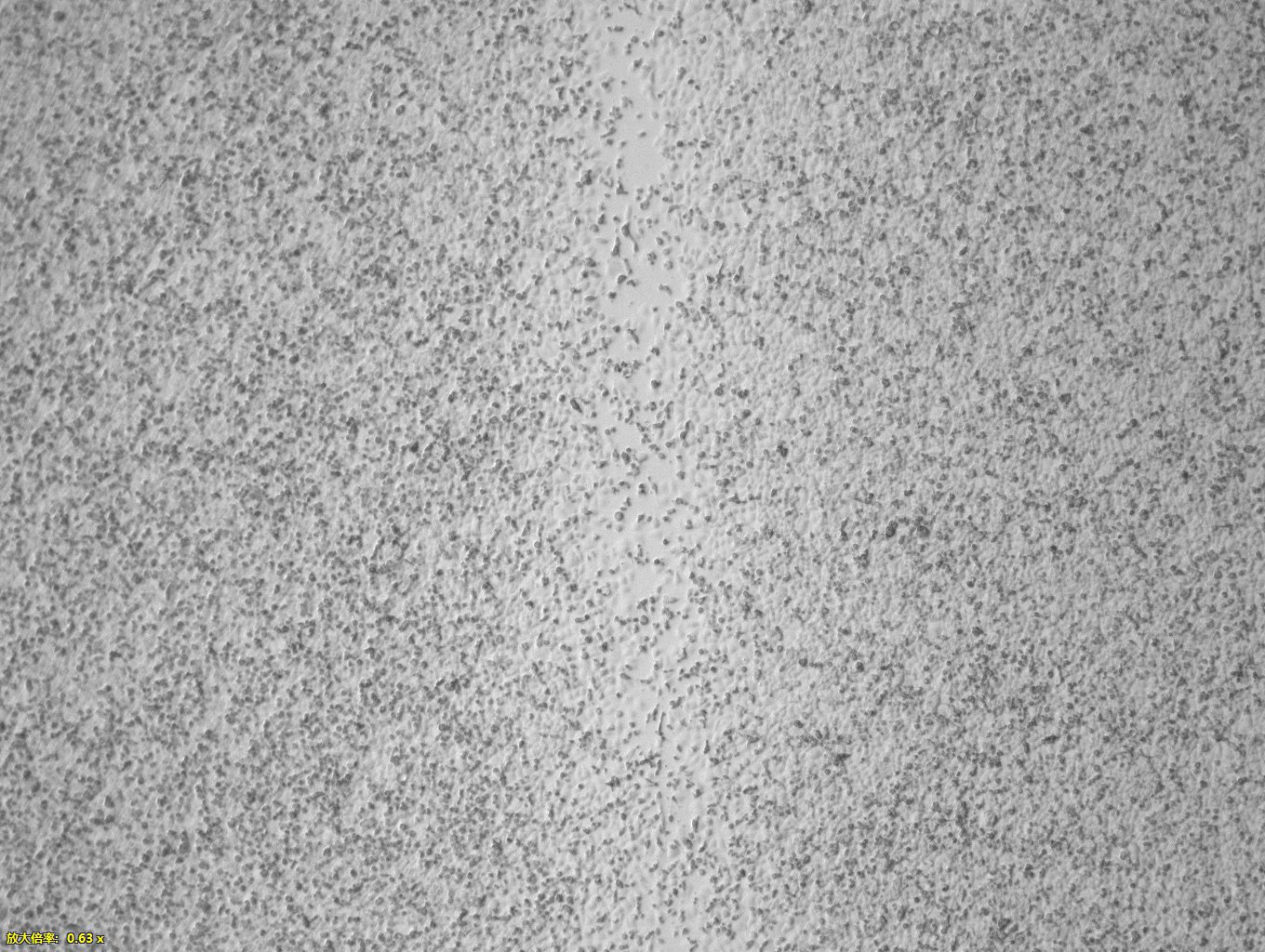

Supplement: Supplementary file 1 [file DataSheet1.zip › 48h/nc-2/图像_66651.jpg]

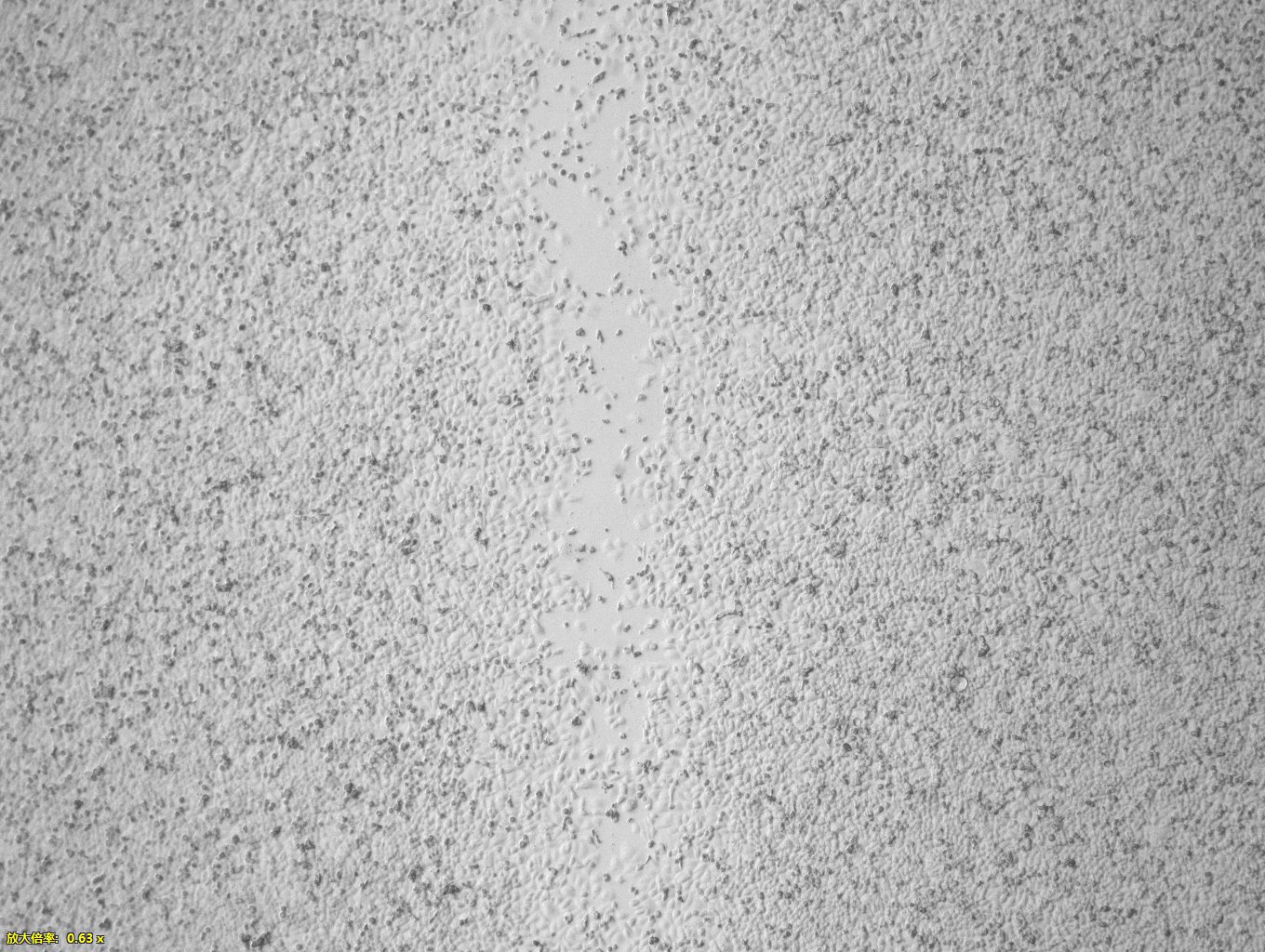

Supplement: Supplementary file 1 [file DataSheet1.zip › 48h/nc-2/图像_66652.jpg]

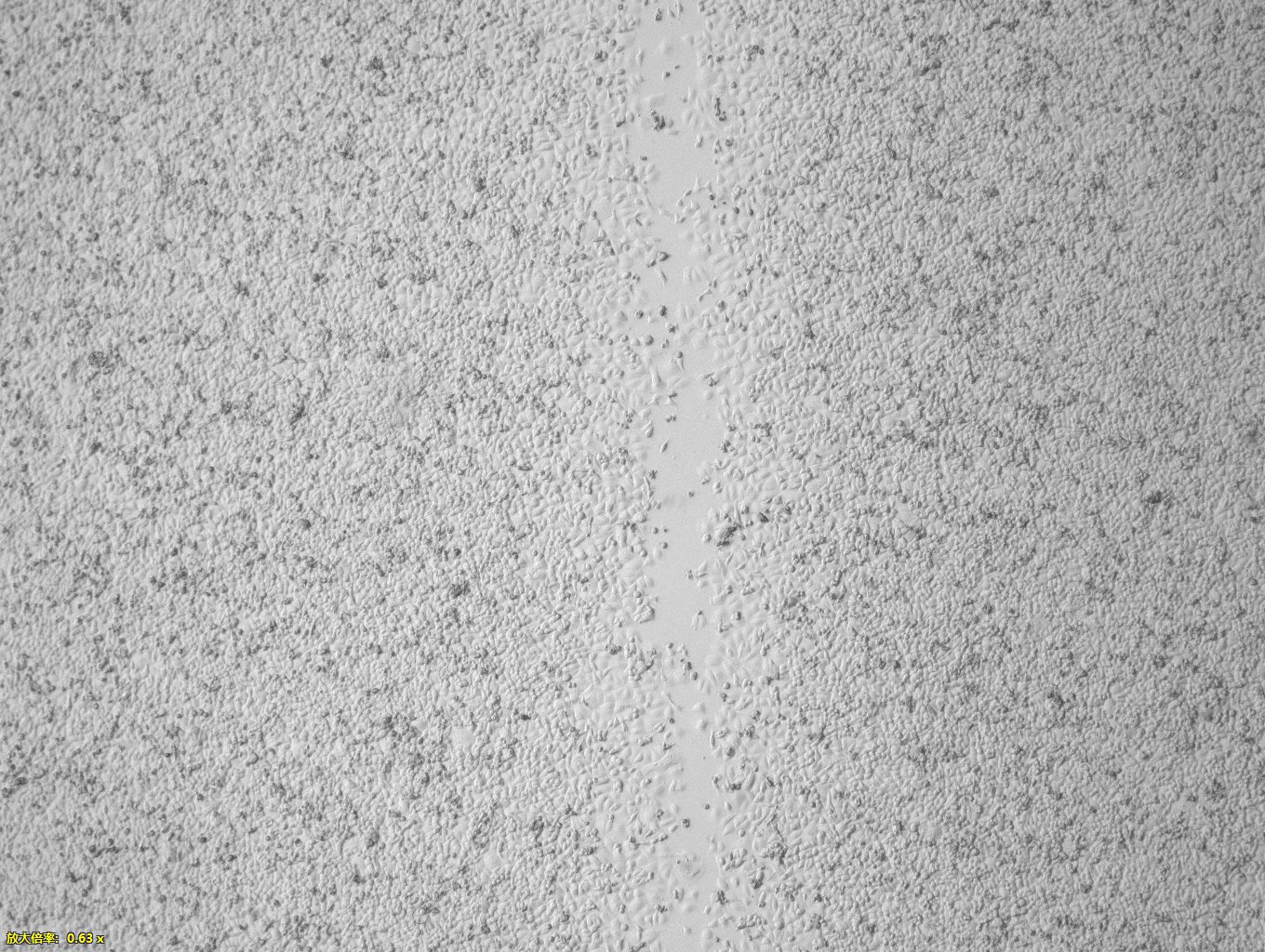

Supplement: Supplementary file 1 [file DataSheet1.zip › 48h/nc-2/图像_66653.jpg]

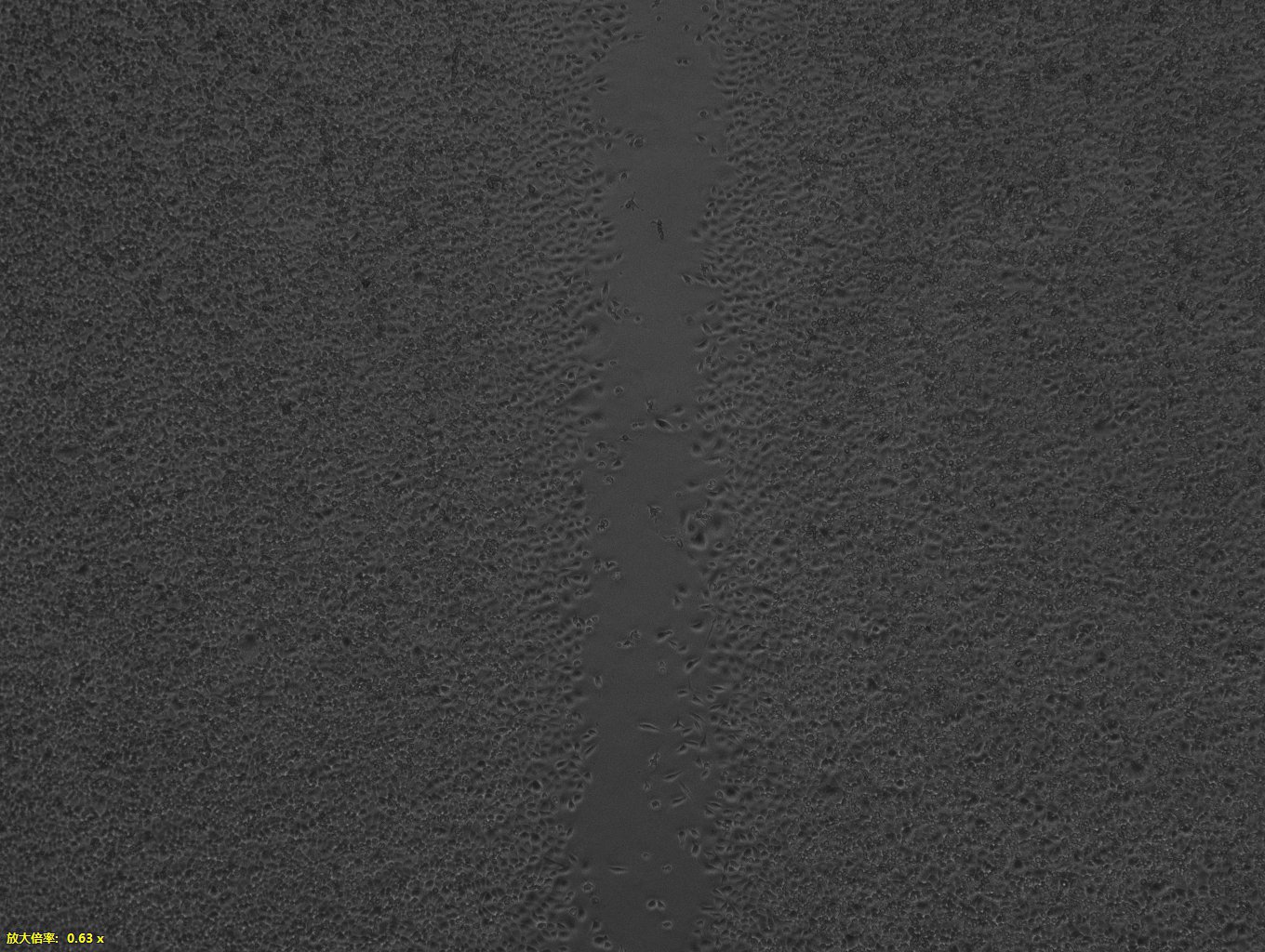

Supplement: Supplementary file 1 [file DataSheet1.zip › 48h/sirna-1/图像_65941.jpg]

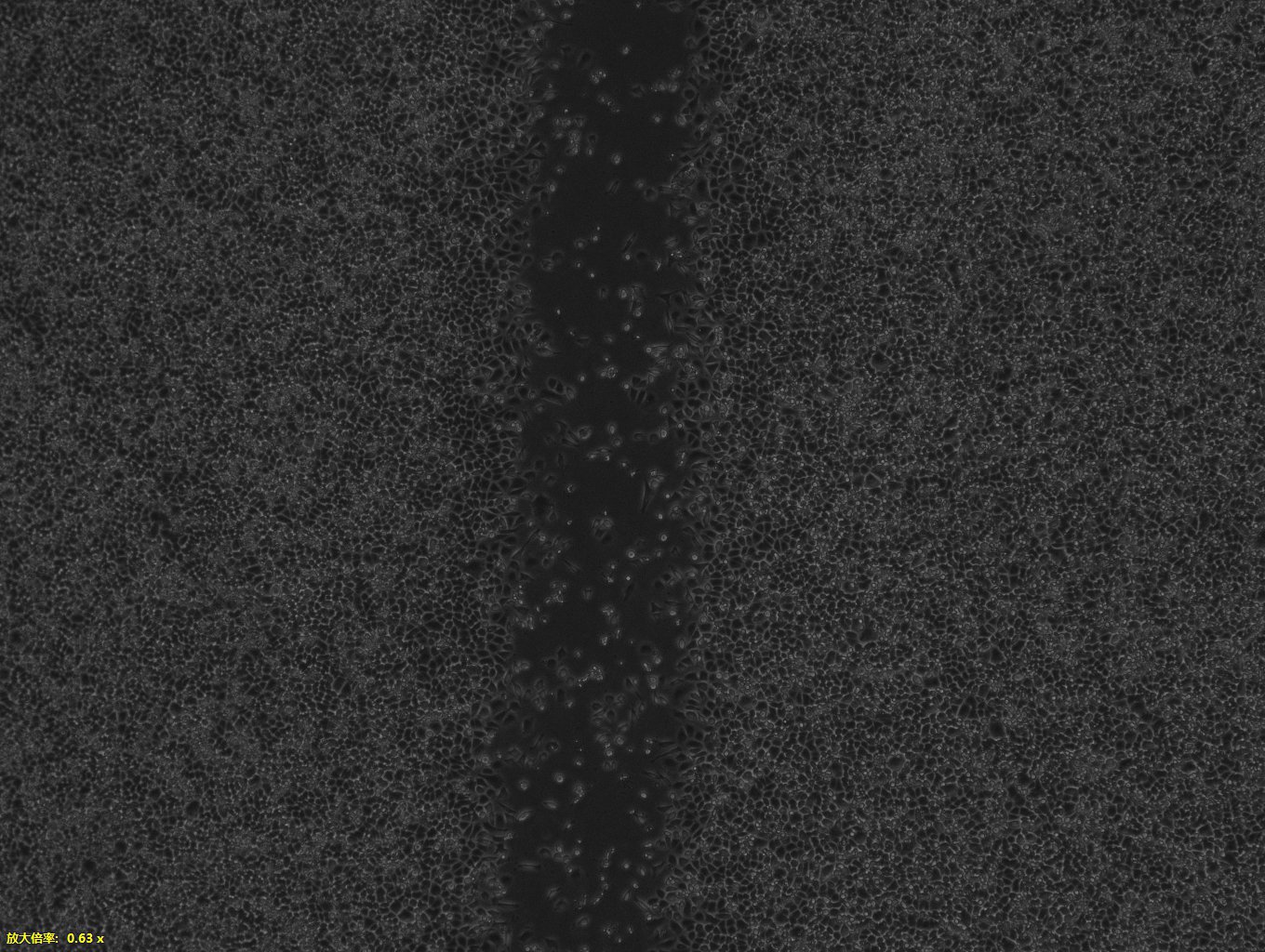

Supplement: Supplementary file 1 [file DataSheet1.zip › 48h/sirna-1/图像_65942.jpg]

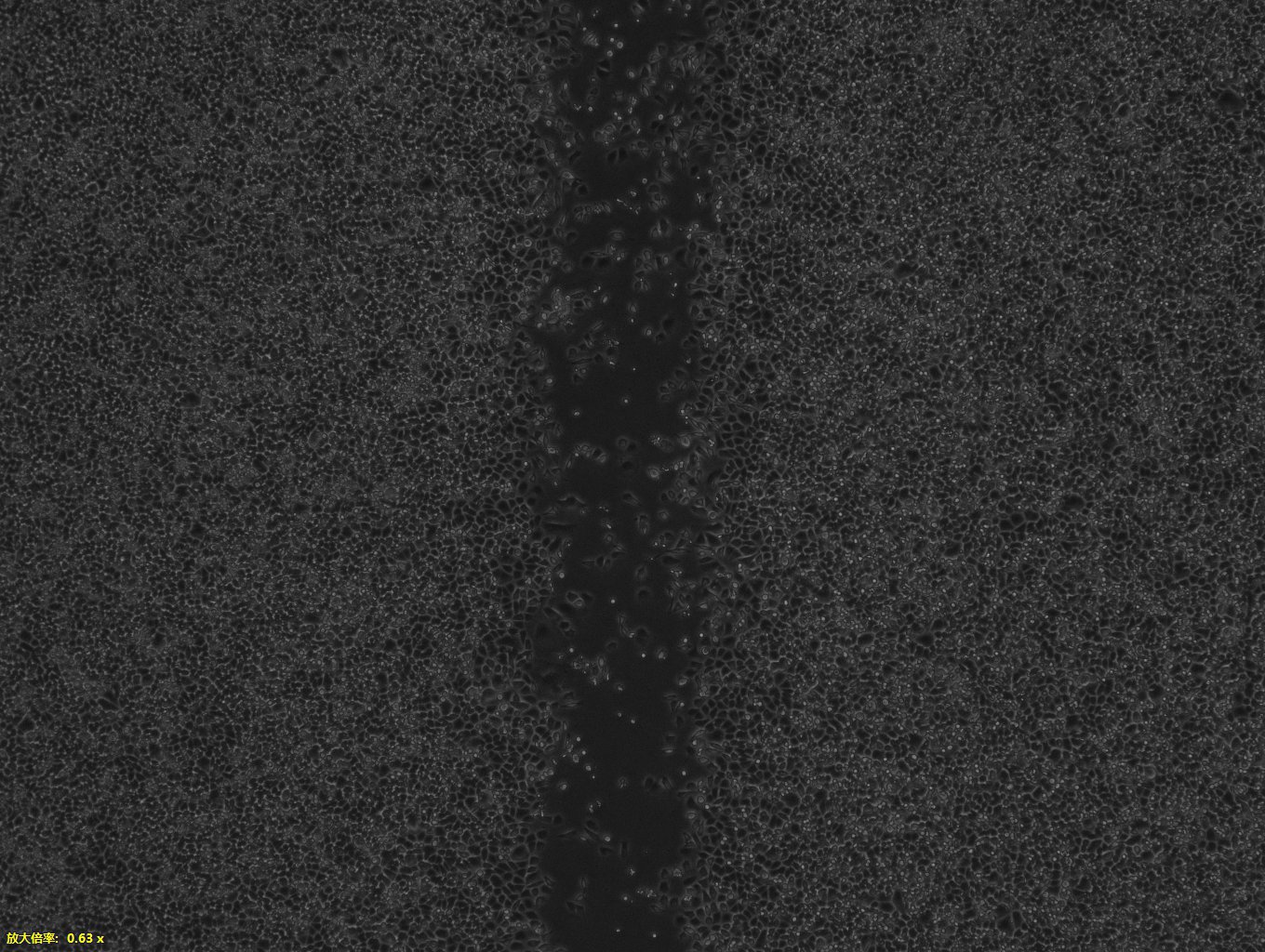

Supplement: Supplementary file 1 [file DataSheet1.zip › 48h/sirna-1/图像_65943.jpg]

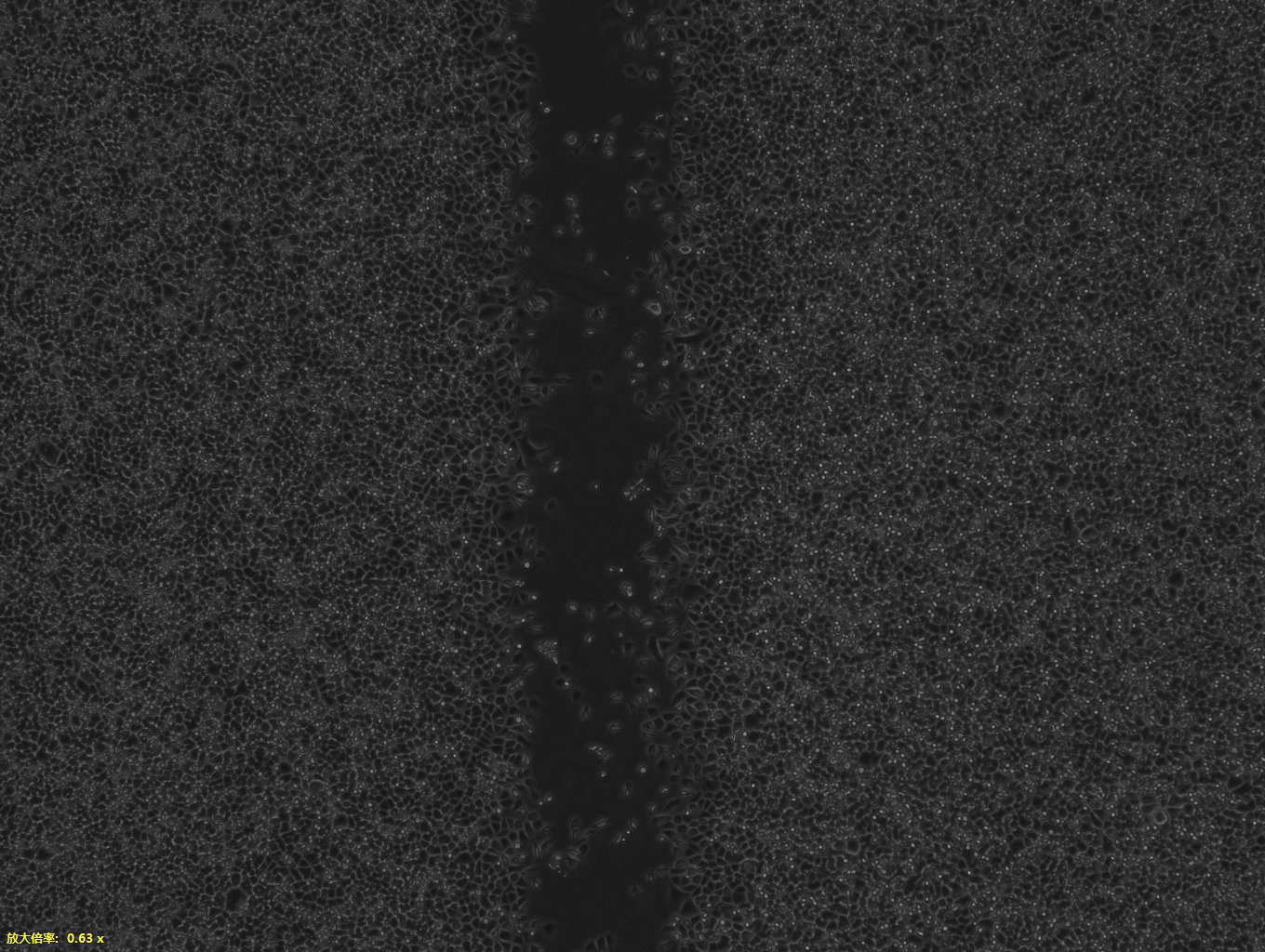

Supplement: Supplementary file 1 [file DataSheet1.zip › 48h/sirna-1/图像_65944.jpg]

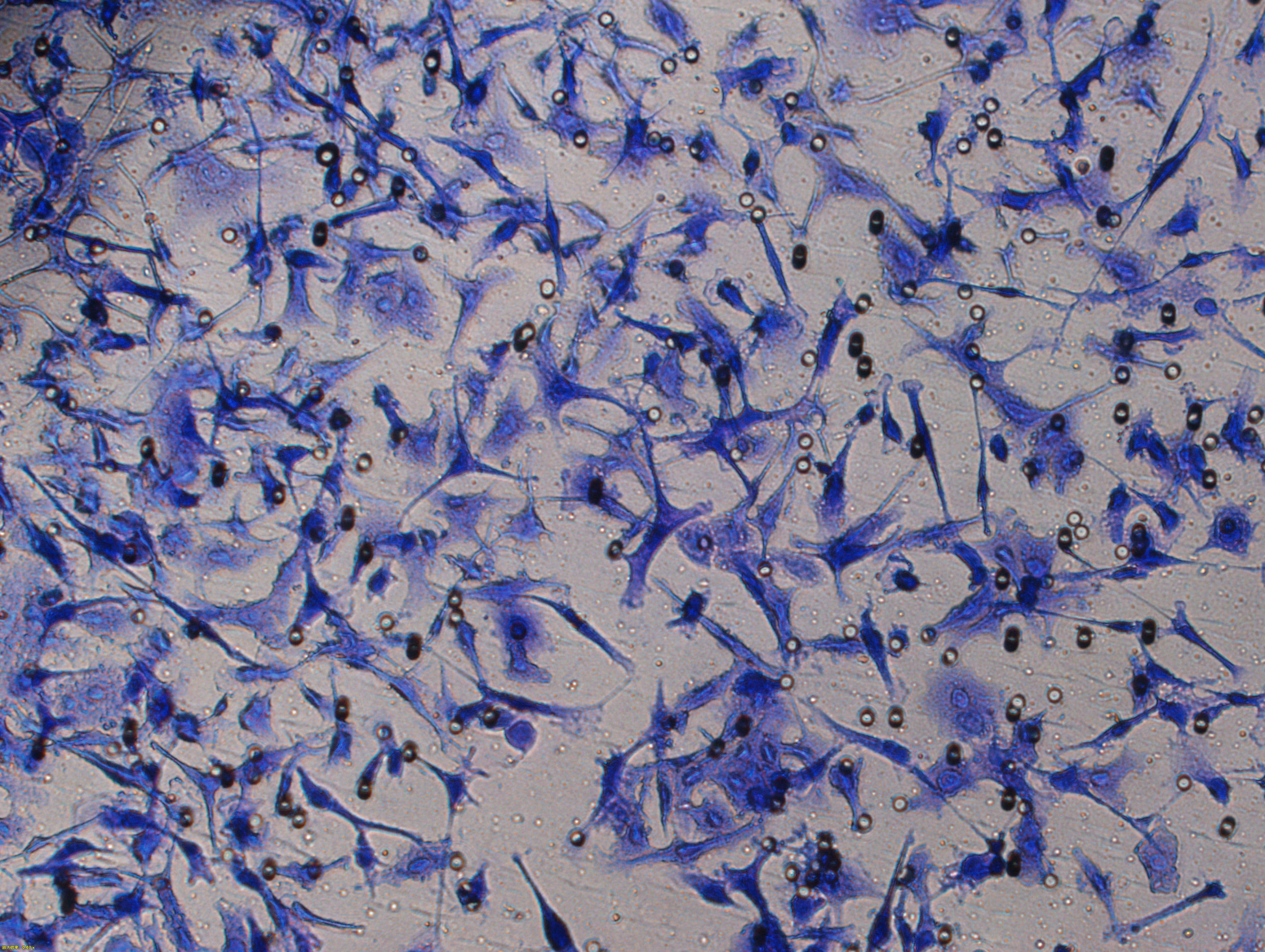

Supplement: Supplementary file 2 [file DataSheet2.zip › invasion/nc/Rep1.jpg]

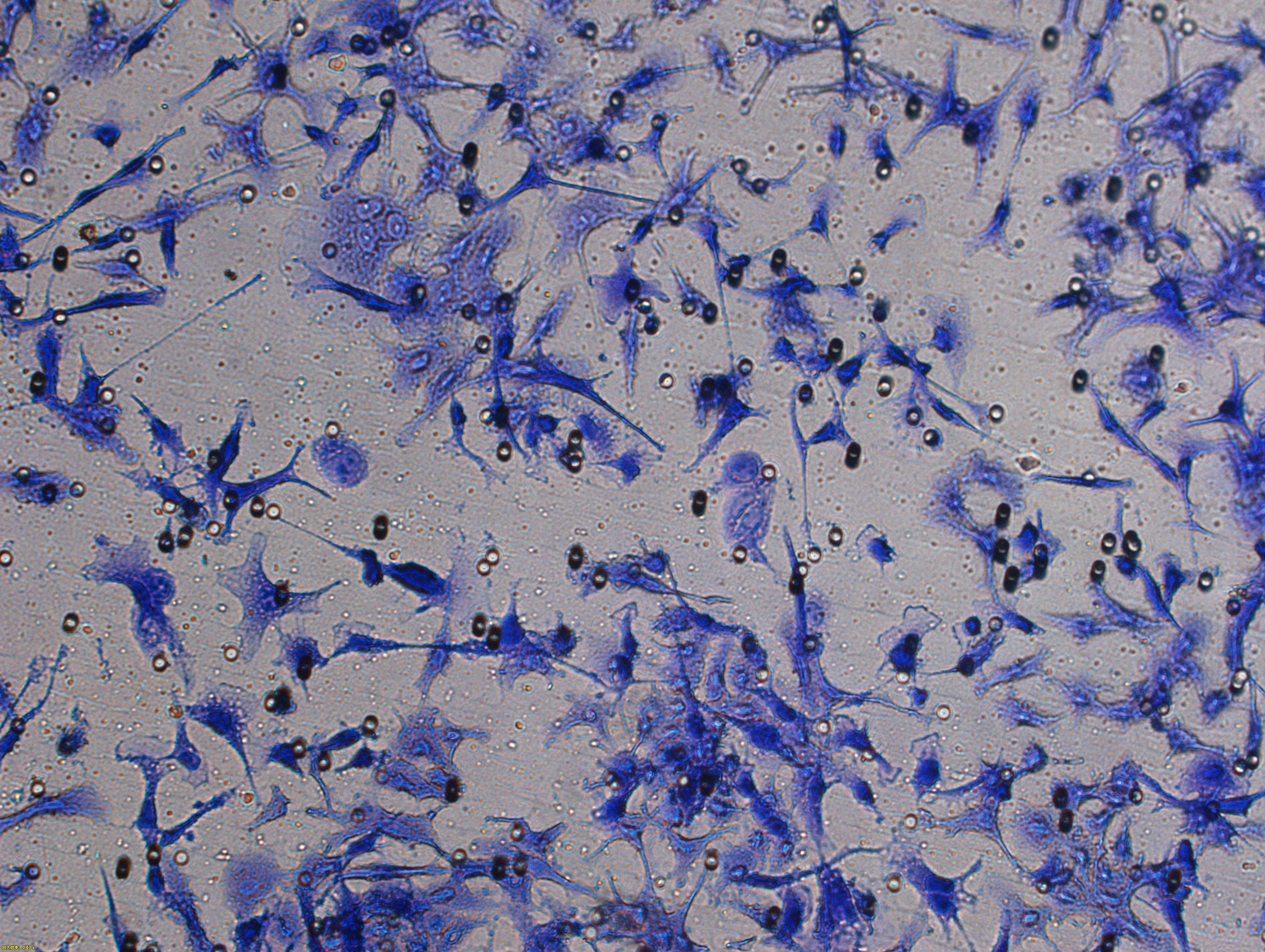

Supplement: Supplementary file 2 [file DataSheet2.zip › invasion/nc/Rep2.jpg]

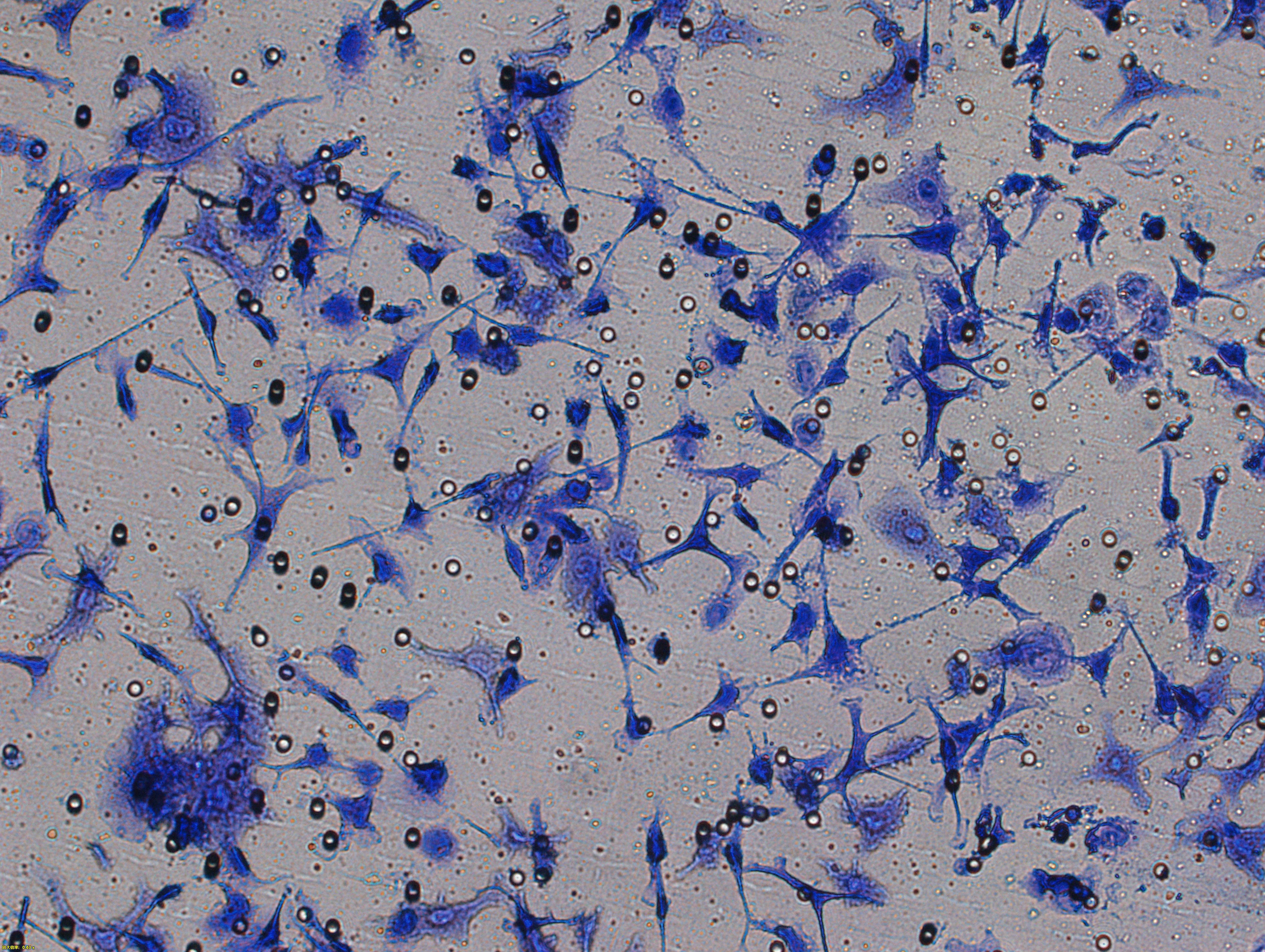

Supplement: Supplementary file 2 [file DataSheet2.zip › invasion/nc/Rep3.jpg]

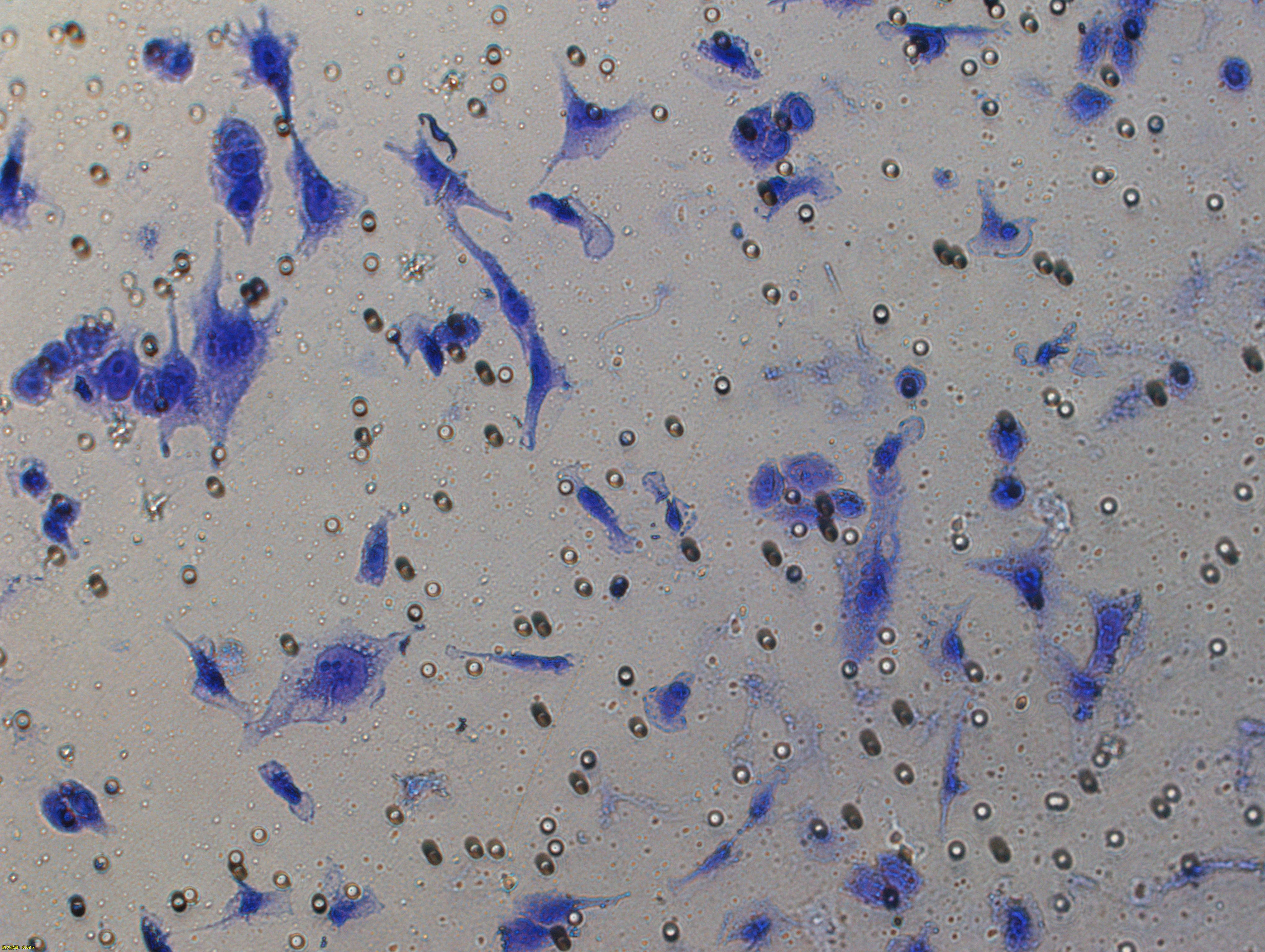

Supplement: Supplementary file 2 [file DataSheet2.zip › invasion/sirna/Rep1.jpg]

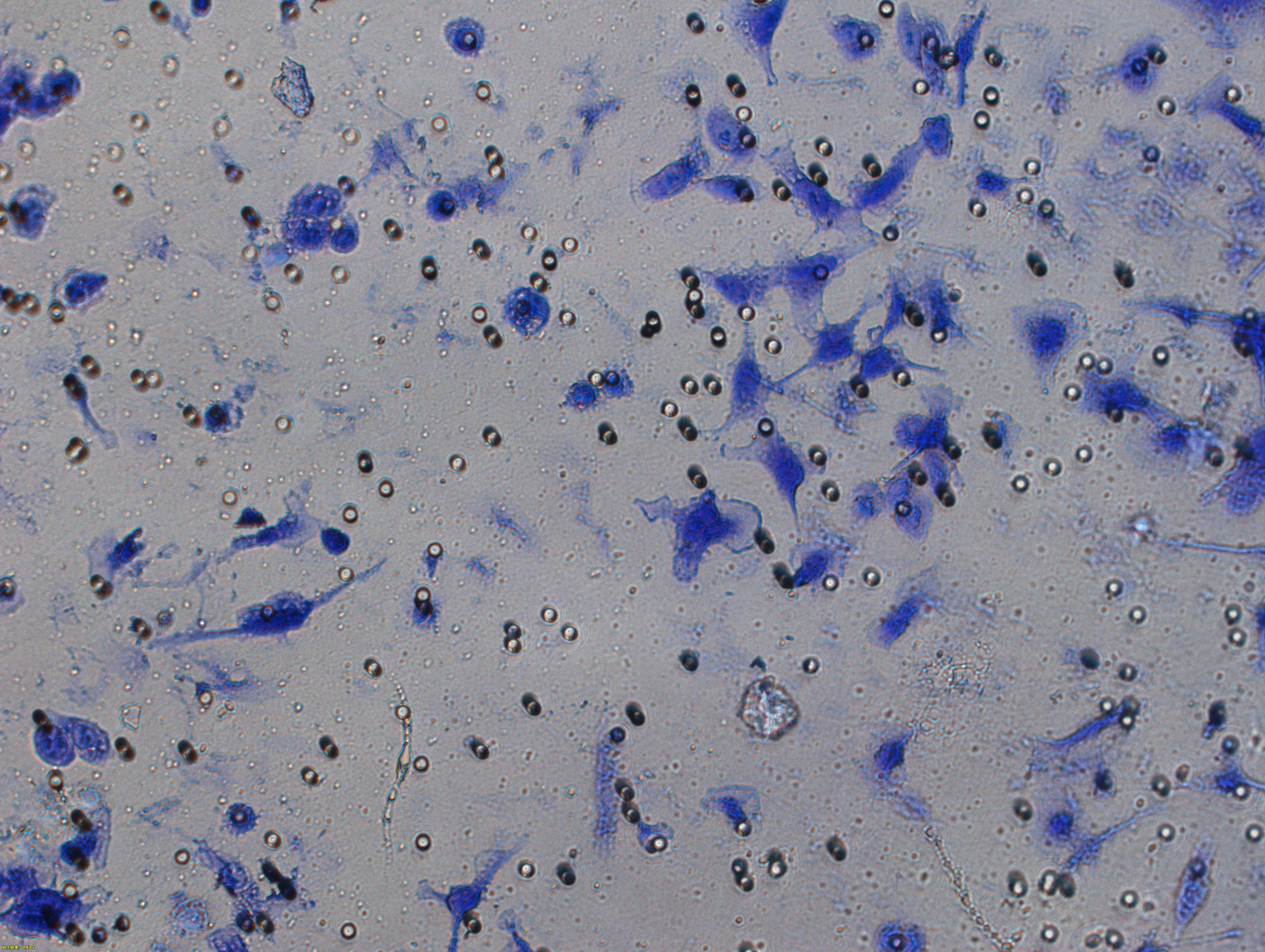

Supplement: Supplementary file 2 [file DataSheet2.zip › invasion/sirna/Rep2.jpg]

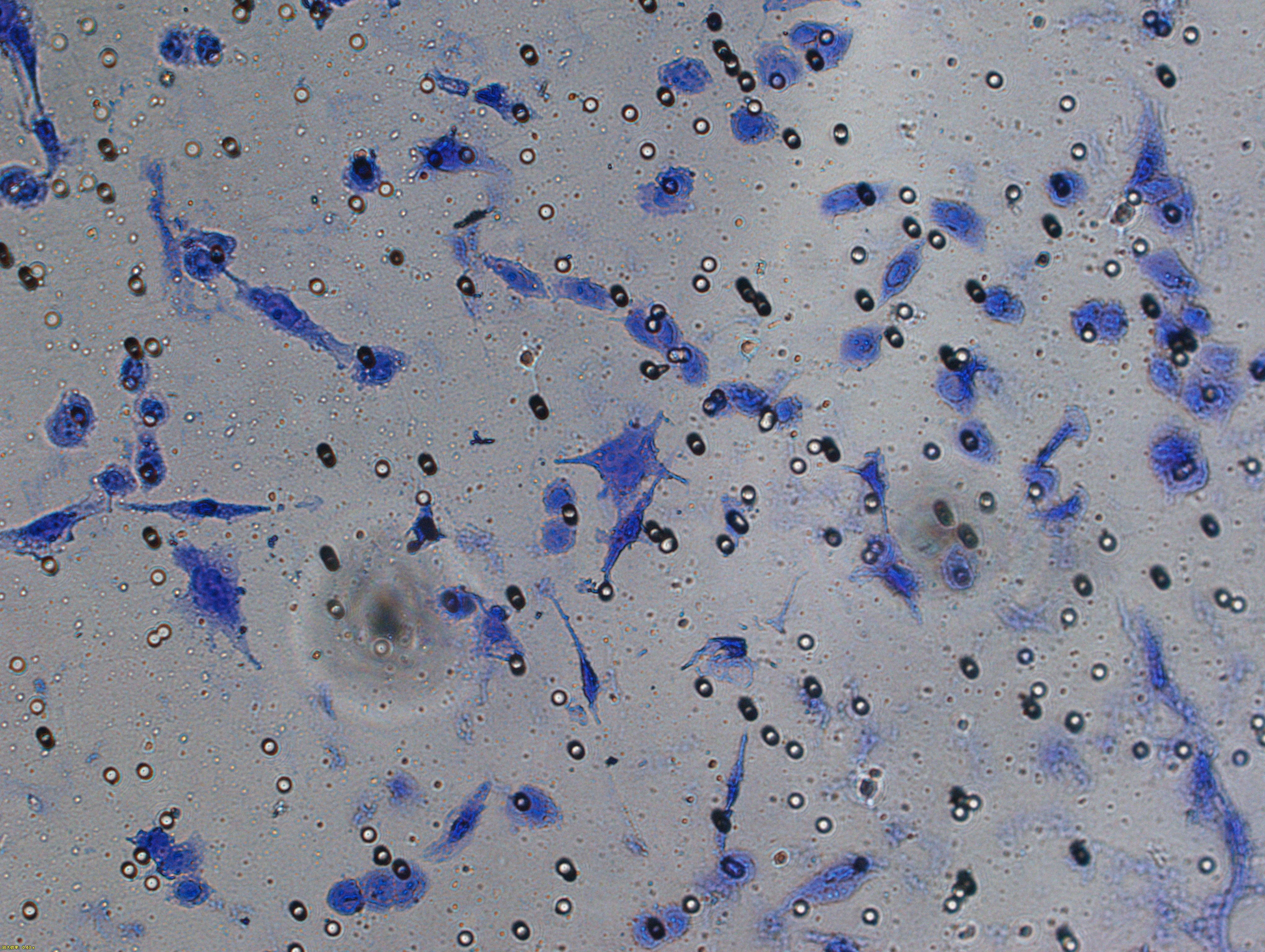

Supplement: Supplementary file 2 [file DataSheet2.zip › invasion/sirna/Rep3.jpg]

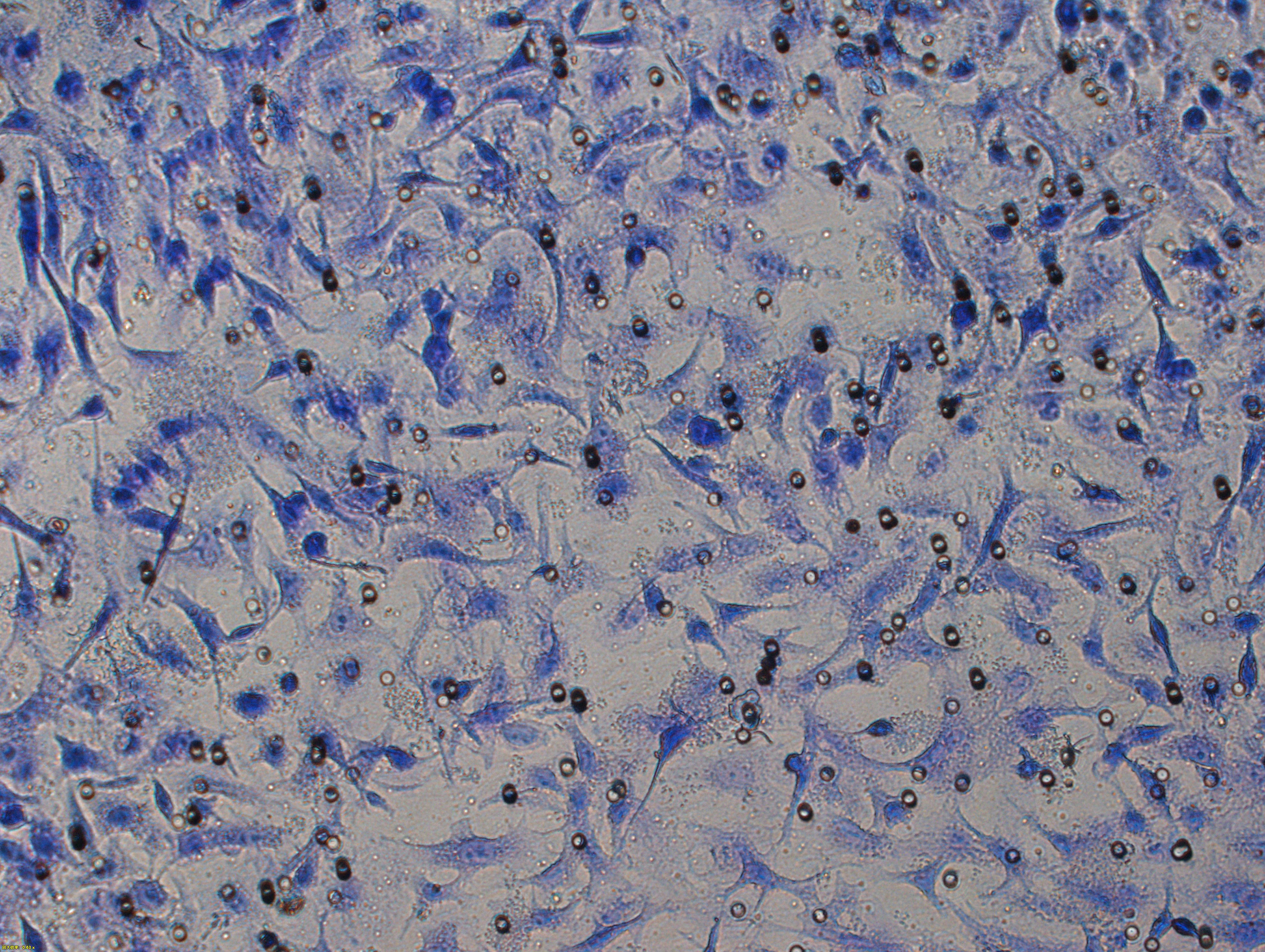

Supplement: Supplementary file 2 [file DataSheet2.zip › Migration/nc/Rep1.jpg]

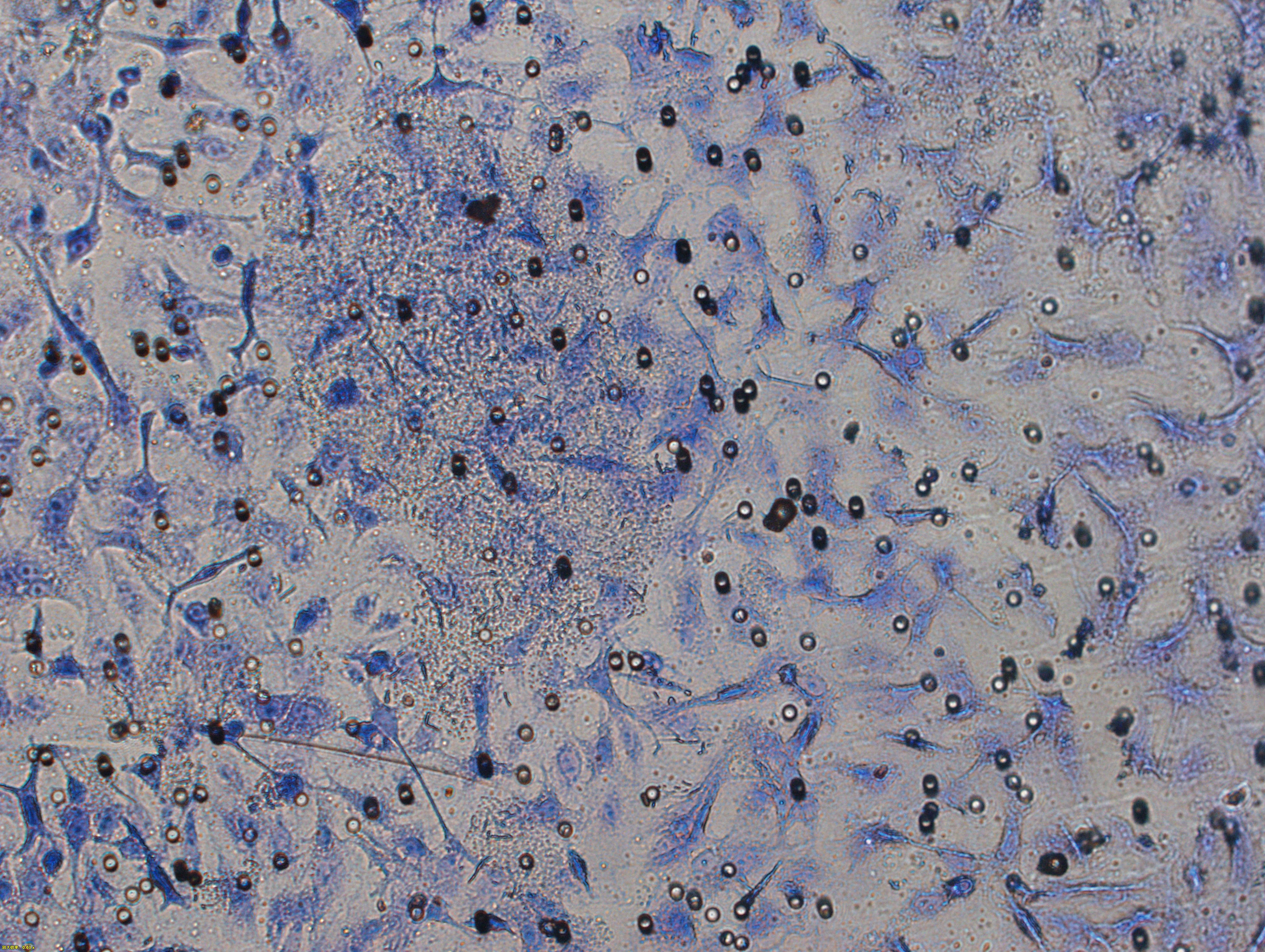

Supplement: Supplementary file 2 [file DataSheet2.zip › Migration/nc/Rep2.jpg]

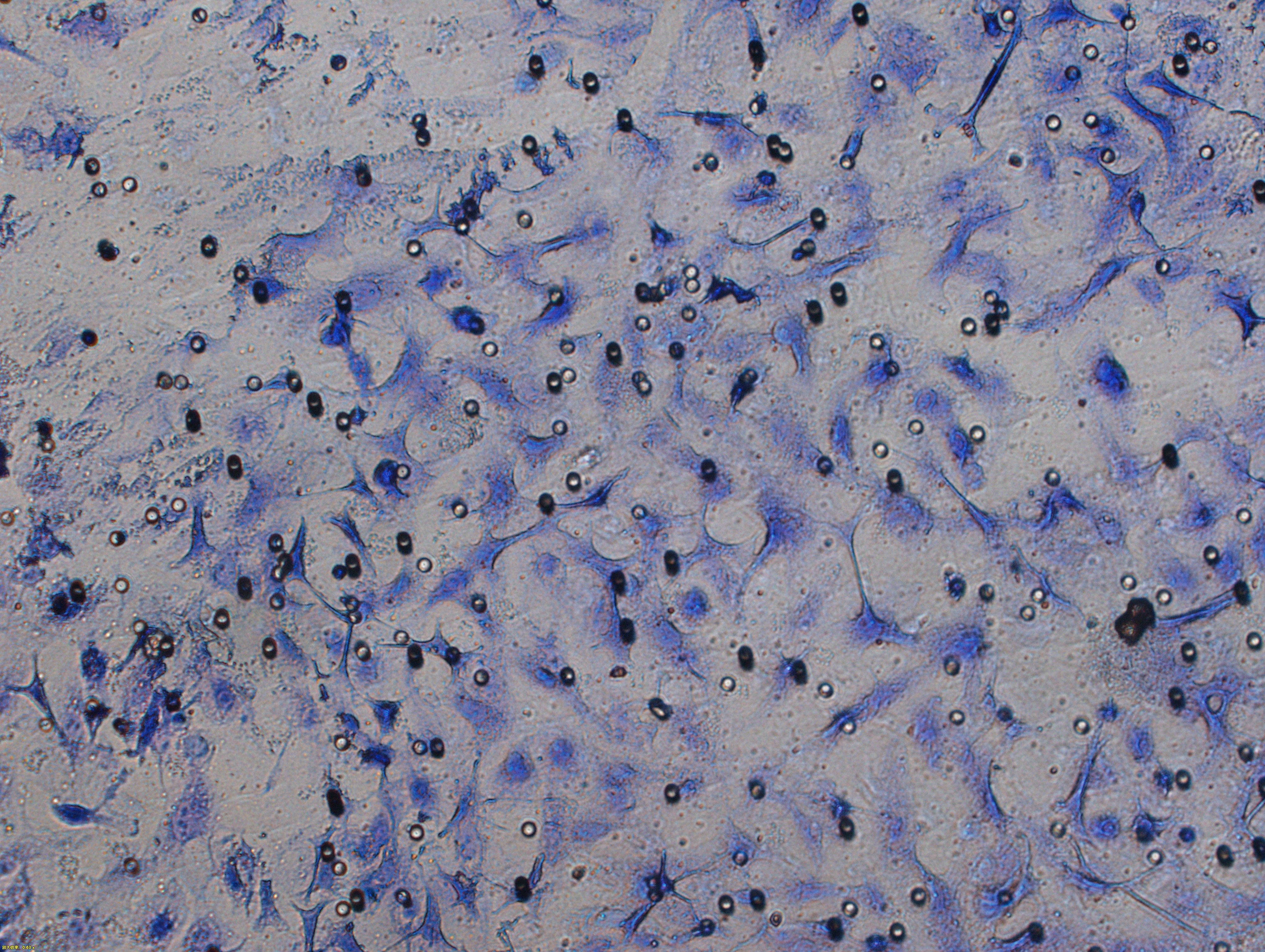

Supplement: Supplementary file 2 [file DataSheet2.zip › Migration/nc/Rep3.jpg]

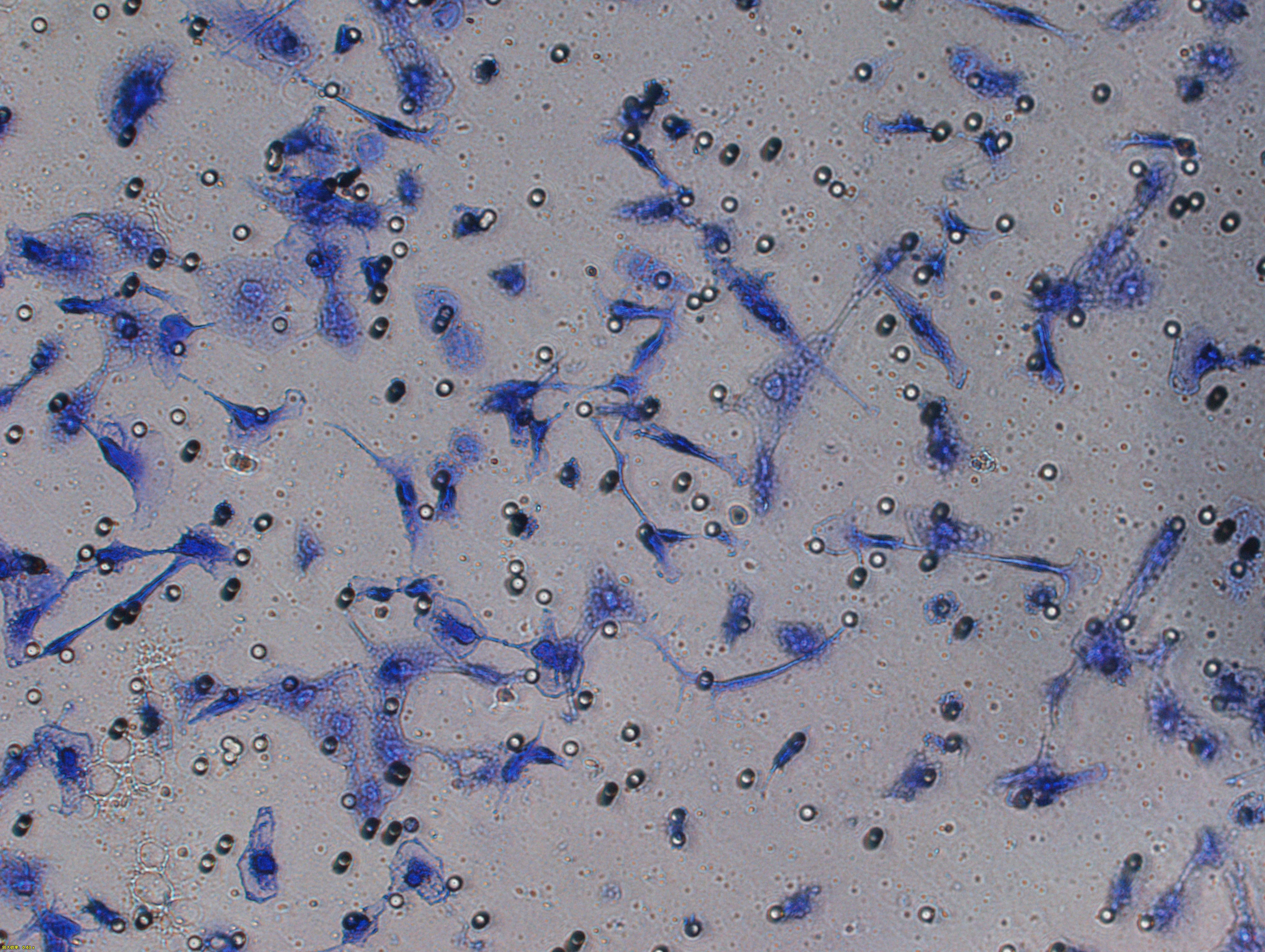

Supplement: Supplementary file 2 [file DataSheet2.zip › Migration/sirna/Rep1.jpg]

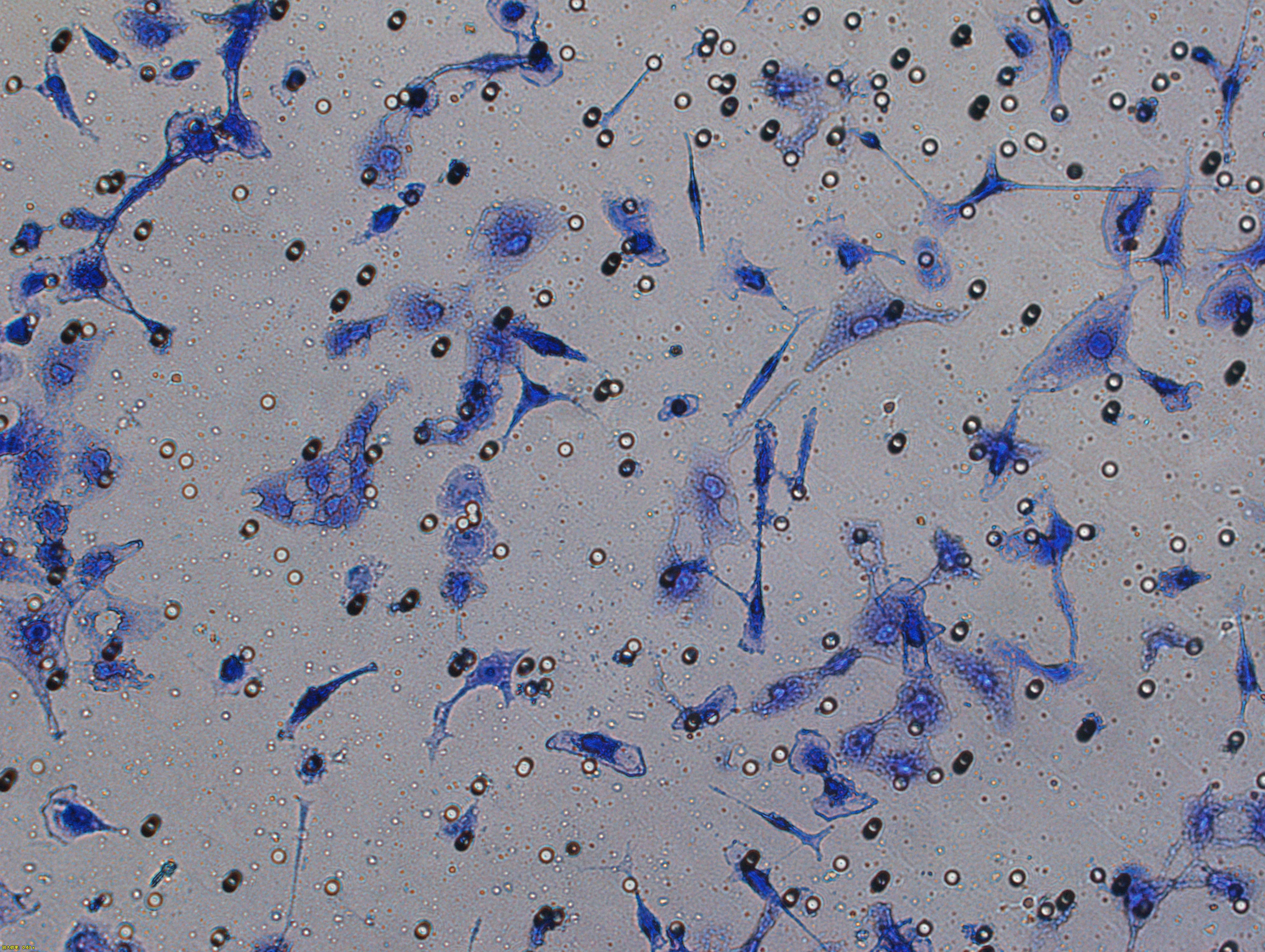

Supplement: Supplementary file 2 [file DataSheet2.zip › Migration/sirna/Rep2.jpg]

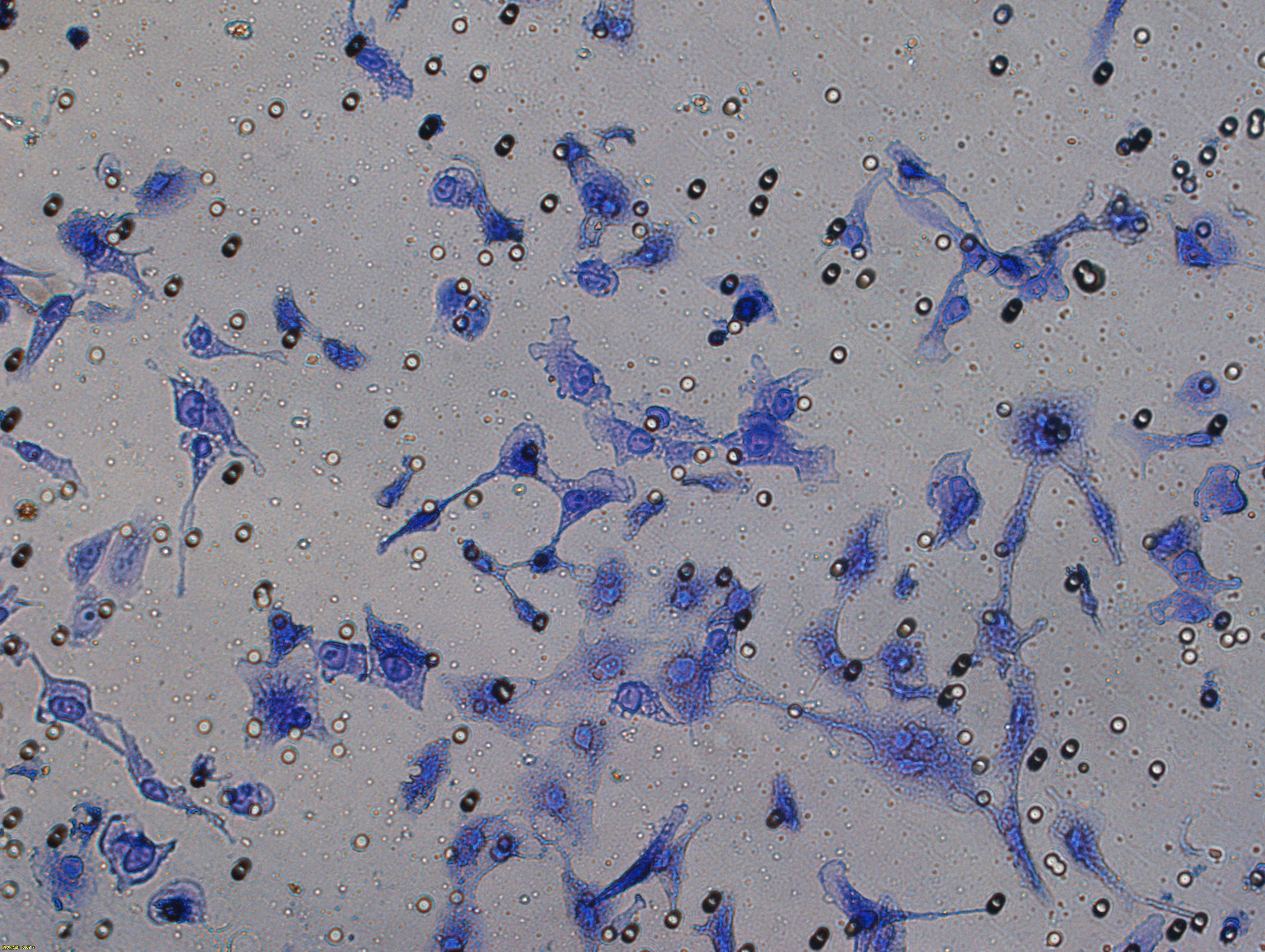

Supplement: Supplementary file 2 [file DataSheet2.zip › Migration/sirna/Rep3.jpg]

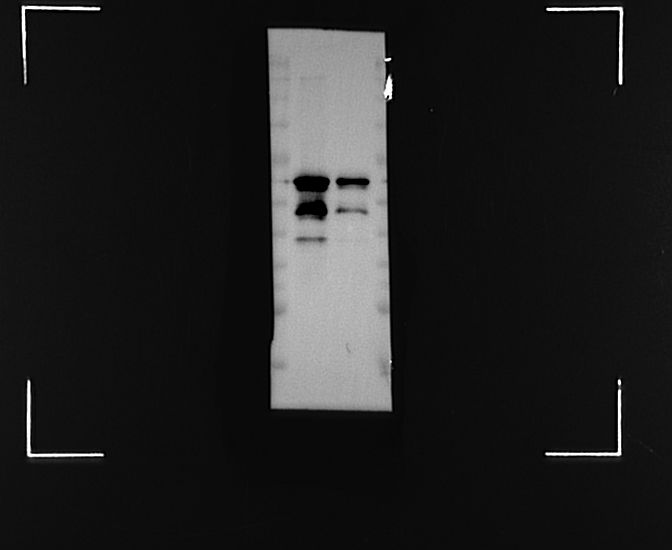

Supplement: Supplementary file 3 [file DataSheet3.zip › CSF1.tif]

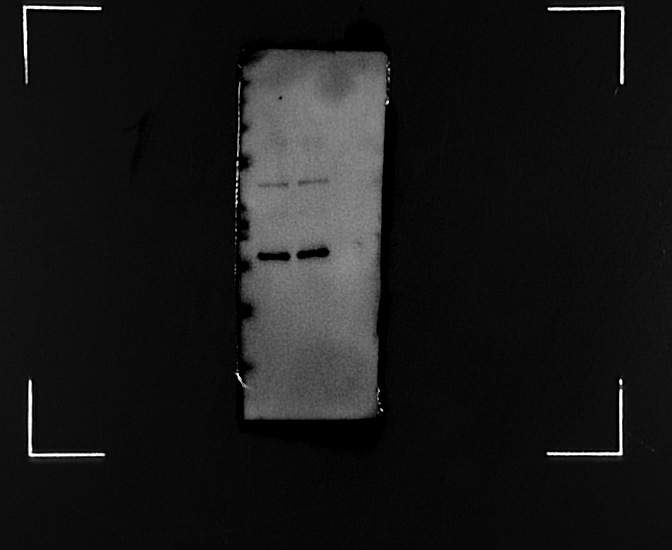

Supplement: Supplementary file 3 [file DataSheet3.zip › GAPDH.tif]

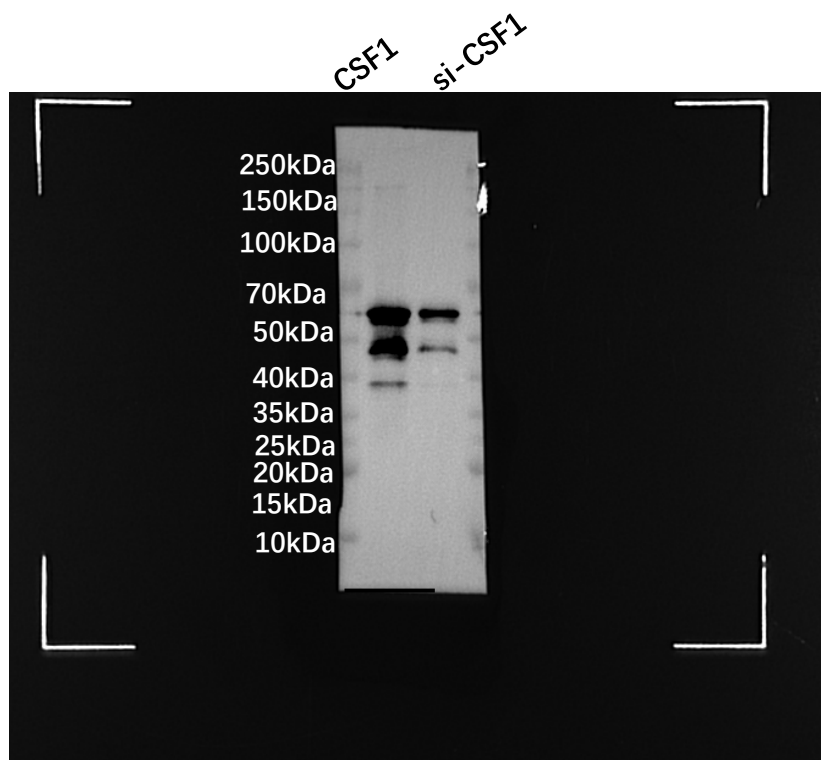

Antibody: CSF1

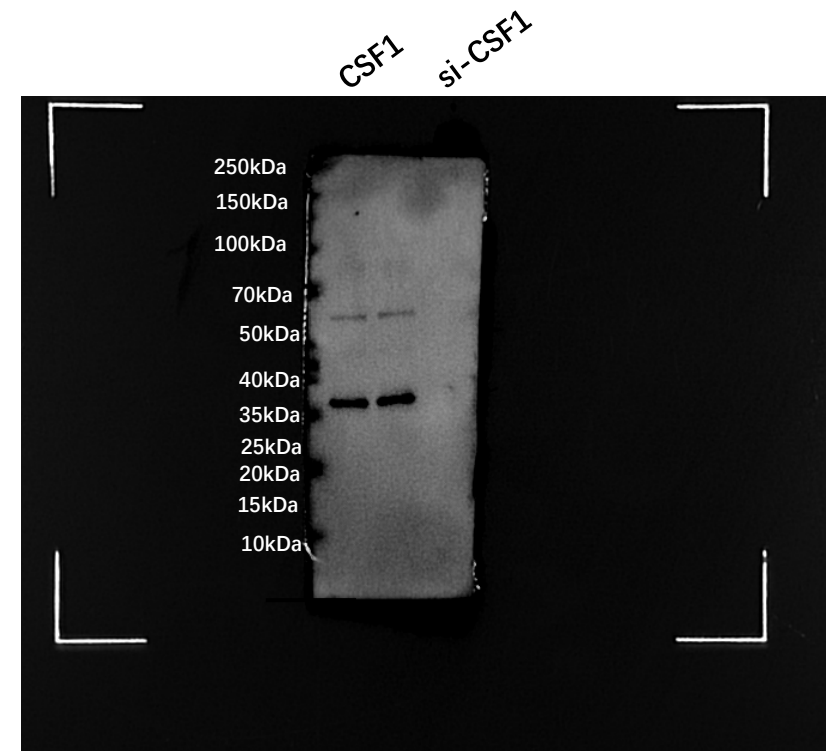

Antibody:GAPDH

Supplement: Supplementary file 3 [file DataSheet3.zip › WB.pdf]
